# MAP4K4 Inhibition Promotes Survival of Human Stem Cell-Derived Cardiomyocytes and Reduces Infarct Size *In Vivo*

**DOI:** 10.1016/j.stem.2019.01.013

**Published:** 2019-04-04

**Authors:** Lorna R. Fiedler, Kathryn Chapman, Min Xie, Evie Maifoshie, Micaela Jenkins, Pelin Arabacilar Golforoush, Mohamed Bellahcene, Michela Noseda, Dörte Faust, Ashley Jarvis, Gary Newton, Marta Abreu Paiva, Mutsuo Harada, Daniel J. Stuckey, Weihua Song, Josef Habib, Priyanka Narasimham, Rehan Aqil, Devika Sanmugalingam, Robert Yan, Lorenzo Pavanello, Motoaki Sano, Sam C. Wang, Robert D. Sampson, Sunthar Kanayaganam, George E. Taffet, Lloyd H. Michael, Mark L. Entman, Tse-Hua Tan, Sian E. Harding, Caroline M.R. Low, Catherine Tralau-Stewart, Trevor Perrior, Michael D. Schneider

**Affiliations:** 1 British Heart Foundation Centre of Research Excellence, National Heart and Lung Institute, Imperial College London, London W12 0NN, UK; 2 Drug Discovery Centre, Department of Medicine, Imperial College London, London SW7 2AZ, UK; 3 Department of Surgery and Cancer, Imperial College London, London W12 0NN, UK; 4 Domainex, Chesterford Research Park, Little Chesterford, Saffron Walden, Essex CB10 1XL, UK; 5 Michael E. DeBakey Heart Center, Department of Medicine, Baylor College of Medicine, Houston, TX 77030, USA; 6 Immunology Research Center, National Health Research Institutes, Zhunan 35053, Taiwan; 7 Department of Pathology and Immunology, Baylor College of Medicine, Houston, TX 77030, USA

**Keywords:** apoptosis, cardiac muscle, drug discovery, heart, signal transduction

## Abstract

Heart disease is a paramount cause of global death and disability. Although cardiomyocyte death plays a causal role and its suppression would be logical, no clinical counter-measures target the responsible intracellular pathways. Therapeutic progress has been hampered by lack of preclinical human validation. Mitogen-activated protein kinase kinase kinase kinase-4 (MAP4K4) is activated in failing human hearts and relevant rodent models. Using human induced-pluripotent-stem-cell-derived cardiomyocytes (hiPSC-CMs) and *MAP4K4* gene silencing, we demonstrate that death induced by oxidative stress requires MAP4K4. Consequently, we devised a small-molecule inhibitor, DMX-5804, that rescues cell survival, mitochondrial function, and calcium cycling in hiPSC-CMs. As proof of principle that drug discovery in hiPSC-CMs may predict efficacy *in vivo*, DMX-5804 reduces ischemia-reperfusion injury in mice by more than 50%. We implicate MAP4K4 as a well-posed target toward suppressing human cardiac cell death and highlight the utility of hiPSC-CMs in drug discovery to enhance cardiomyocyte survival.

## Highlights

• Human iPSC-cardiomyocytes were used for MAP4K4 target validation and drug discovery
• MAP4K4 shRNA protects hiPSC-cardiomyocytes from lethal oxidative stress
• MAP4K4 inhibitors promote hiPSC-cardiomyocyte survival and function
• MAP4K4 inhibition markedly reduces cardiac ischemia-reperfusion injury in mice

## Graphical Abstract

**Figure:**
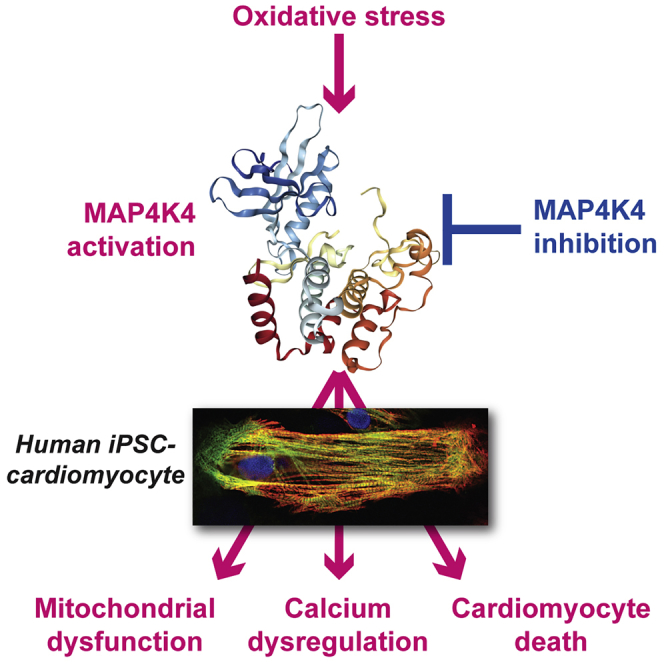

## Teaser

Using human iPSC-derived cardiomyocytes to enhance cardiac drug discovery, Fiedler et al. performed MAP4K4 target validation by gene silencing in this human model. MAP4K4 inhibitors augment human cardiomyocyte viability and function in 2D culture and 3D engineered heart tissue. An exemplar successfully reduces infarct size in proof-of-principle studies in mice.

## Introduction

Heart disease remains the single most common cause of death and disability worldwide and is projected to increase as the population ages, its socio-economic burden consequently rising for the foreseeable future (GBD 2016 Causes of Death Collaborators, 2017, GBD 2016 DALYs and HALE Collaborators, 2017). Cardiac muscle cell death is an instrumental component of both acute ischemic injury and also chronic heart failure, driving dysfunction of the heart as a biomechanical pump (Dorn, 2009, Fiedler et al., 2014, Whelan et al., 2010). To date, however, few human trials for heart disease seek to enhance cardiomyocyte survival directly, and nearly all strategies for cardioprotection have failed between phases I and III, due to the lack of efficacy (Hausenloy and Yellon, 2015, Heusch, 2013, Lincoff et al., 2014, Newby et al., 2014, Ottani et al., 2016, Piot et al., 2008). In 2018 alone, the US Food and Drug Administration approved 13 drugs for cancer, yet none for cardiac indications (US Food and Drug Administration, 2018). Among other recognized limitations, the conventional pipeline for cardiac drug development lacks any human preclinical model whatsoever for testing proposed counter-measures (Fordyce et al., 2015, Gromo et al., 2014). The routine cardiac platforms for predicting efficacy are inherently flawed, and the creation of novel approvable medicines is “dead” or “hibernating” (Fordyce et al., 2015). In contrast, for cancer therapeutics, the systematic use of representative human cell lines is instrumental to preclinical evaluation of tumor responses, fueling innovation (Wilding and Bodmer, 2014). By offering analogous, scalable, routine access to human cardiac biology, cardiomyocytes derived from human induced pluripotent stem cells (hiPSC-CMs) have gained wide acceptance as transformative in addressing this unmet need, predicting cardiotoxicity, modeling patient-specific pathways, and enhancing cardiac target validation and drug discovery as a partial but auspicious means to improve the *in vitro* surrogates (Bellin et al., 2012, Blinova et al., 2018, Burridge et al., 2016, Cameron et al., 2013, Gintant et al., 2017, Lee et al., 2017b, Liang et al., 2013, Matsa et al., 2014, Matsa et al., 2016, Sharma et al., 2017).

In preclinical models, the molecular and genetic dissection of cardiac cell death suggests potential nodal control points, among them, signaling pathways mediated by mitogen-activated protein kinases (MAPKs), especially Jun N-terminal kinase (JNK) and p38 (Dorn, 2009, Fiedler et al., 2014, Whelan et al., 2010). Because the “terminal” MAPKs p38 and JNK receive inputs from multiple signals, both protective and adverse, it is logical to consider targeting specific proximal kinases that might couple these to cell death more selectively. MAP kinase kinase kinase kinases (MAP4Ks) are the most proximal protein kinases in the MAPK superfamily. MAP4K4 (HPK/GCK-like kinase [HGK]; NCK-interacting kinase [NIK]) is a serine-threonine kinase related to Ste20 in *S. cerevisiae* (Su et al., 1997). Like their yeast ortholog, the mammalian Ste20 kinases control cell motility, fate, proliferation, and stress responses (Dan et al., 2001). Deleting MAP4K4 in mice is embryonic lethal, owing to cell motility defects during mesoderm patterning (Xue et al., 2001), functions that are conserved in *Drosophila* and *C. elegans* (Chapman et al., 2008) but obscure its possible function in adult biology. With the cloning of human MAP4K4 came the first such evidence, coupling pro-inflammatory cytokines to JNK (Yao et al., 1999). MAP4K4 is now appreciated as a pivotal mediator of inflammation, cytoskeletal function, and, notably, cell death, with well-established contributions to cancer, diabetes, and neurodegeneration (Chen et al., 2014, Larhammar et al., 2017, Lee et al., 2017a, Miled et al., 2005, Vitorino et al., 2015, Yang et al., 2013, Yue et al., 2014).

Presently, MAP4K4 function in the heart is conjectural, but a pathobiological role is suggested by its engagement of transforming-growth-factor-β-activated kinase-1 (TAK1/MAP3K7), JNK, and p38 MAPK (Yao et al., 1999, Zohn et al., 2006), three downstream MAPKs with reported pro-death functions in cardiac muscle cells (Fiedler et al., 2014, Jacquet et al., 2008, Zhang et al., 2000). Consequently, in prioritizing among proximal MAPKs as the basis for a novel small-molecule program, we chose MAP4K4 as a logical starting point. Here, using hiPSC-CMs as a human platform for more relevant target validation and compound development, we demonstrate MAP4K4 to be a druggable target in human cardiac injury. We devised highly selective pharmacological inhibitors of MAP4K4, demonstrate that inhibiting MAP4K4 effectively protects human cardiomyocytes from lethal experimental injury, and take an exemplar forward from human cardiomyocytes into further proof-of-concept studies in mice.

## Results

### MAP4K4 Is Activated by Cardiac Death Signals and Promotes Cardiac Muscle Cell Death

To ascertain the scientific case for inhibiting MAP4K4 in cardiac cell death, three biological settings first were explored: diseased human heart tissue; mouse models; and rat cardiomyocytes (Figures S1–S4). Activation of human cardiac MAP4K4 was prevalent in chronic heart failure from diverse etiologies, associated with active (cleaved) caspase-3, a mediator of apoptosis (Figure S1A), and activation of the MAP3K intermediary, TAK1 (Figure S1B), which itself can drive cardiac cell death (Zhang et al., 2000). Likewise, in adult mouse myocardium (Figure S1C) and cultured rat cardiomyocytes (Figure S1D), MAP4K4 was activated by clinically relevant provocations that promote cardiac muscle cell death, including ischemia-reperfusion injury and H_2_O_2_ as the oxidative stress, a pathobiological hallmark of acute and chronic cardiac disorders (Bertero and Maack, 2018, Brown and Griendling, 2015). Next, we simulated this increase in MAP4K4 activity by viral gene transfer in rat ventricular myocytes (Figure S2). A pro-apoptotic effect of catalytically active MAP4K4 was confirmed (Figures 2A and 2B), potentially involving TAK1 (Figures S2C and S2D), JNK (Figures S2A, S2D, and S2E), and the mitochondrial death pathway (Figures S2F and S2G). In adult mice, cardiomyocyte-restricted *MAP4K4* sensitized the myocardium to otherwise sub-lethal death signals, potentiating myocyte loss, fibrosis, and dysfunction (*Myh6-Gnaq*: Figures S3A–S3D; increased workload: Figures S3E–S3H and Table S1). Conversely, cultured rat cardiomyocytes were protected at least 50% by kinase-dead MAP4K4 (Figure S4A) or *MAP4K4* short hairpin RNA (shRNA) (Figures S4B–S4D). Together, these gain-of-function, dominant-negative, and loss-of-function studies suggest a pivotal role for MAP4K4 in cardiac muscle cell death, albeit with the diverse limitations inherent to non-human models.

### MAP4K4 Target Validation in Human Stem Cell-Derived Cardiomyocytes

To establish whether an equivalent requirement for MAP4K4 also exists in human cardiac muscle cells, we investigated its role in cardiomyocytes derived from human induced pluripotent stem cells. Human iPSC-CMs are available more readily and in higher number than myocytes from clinical biopsies or explanted human hearts, have been deployed successfully as *in vitro* models of hereditary heart disorders (Birket et al., 2015, Hinson et al., 2015, Moretti et al., 2010, Yazawa et al., 2011), have predictive power regarding arrhythmic susceptibility (Gintant et al., 2017), and have begun to yield insights into cell death in drug-induced and hereditary cardiomyopathies (Burridge et al., 2016, Cameron et al., 2013, Lee et al., 2017b, Liang et al., 2013, Lin et al., 2015, Matsa et al., 2016, Sharma et al., 2017). For this constellation of reasons, hiPSC-CMs are envisioned as a highly auspicious tool for cardiac drug discovery (Devalla et al., 2015, Gintant et al., 2017, Kirby et al., 2018, Matsa et al., 2014). Their potential, however, has largely been unheeded for drug creation in ischemic heart disease, the most prevalent of all cardiac disorders (GBD 2016 Causes of Death Collaborators, 2017, GBD 2016 DALYs and HALE Collaborators, 2017). We chose to test MAP4K4 function in this clinical context, using well-characterized, purified, commercially available hiPSC-CMs that have gained acceptance by industry and regulatory authorities as highly indicative of drug safety in humans (Blinova et al., 2018, Gintant et al., 2017, Magdy et al., 2018, Sala et al., 2017).

**Figure 1:**
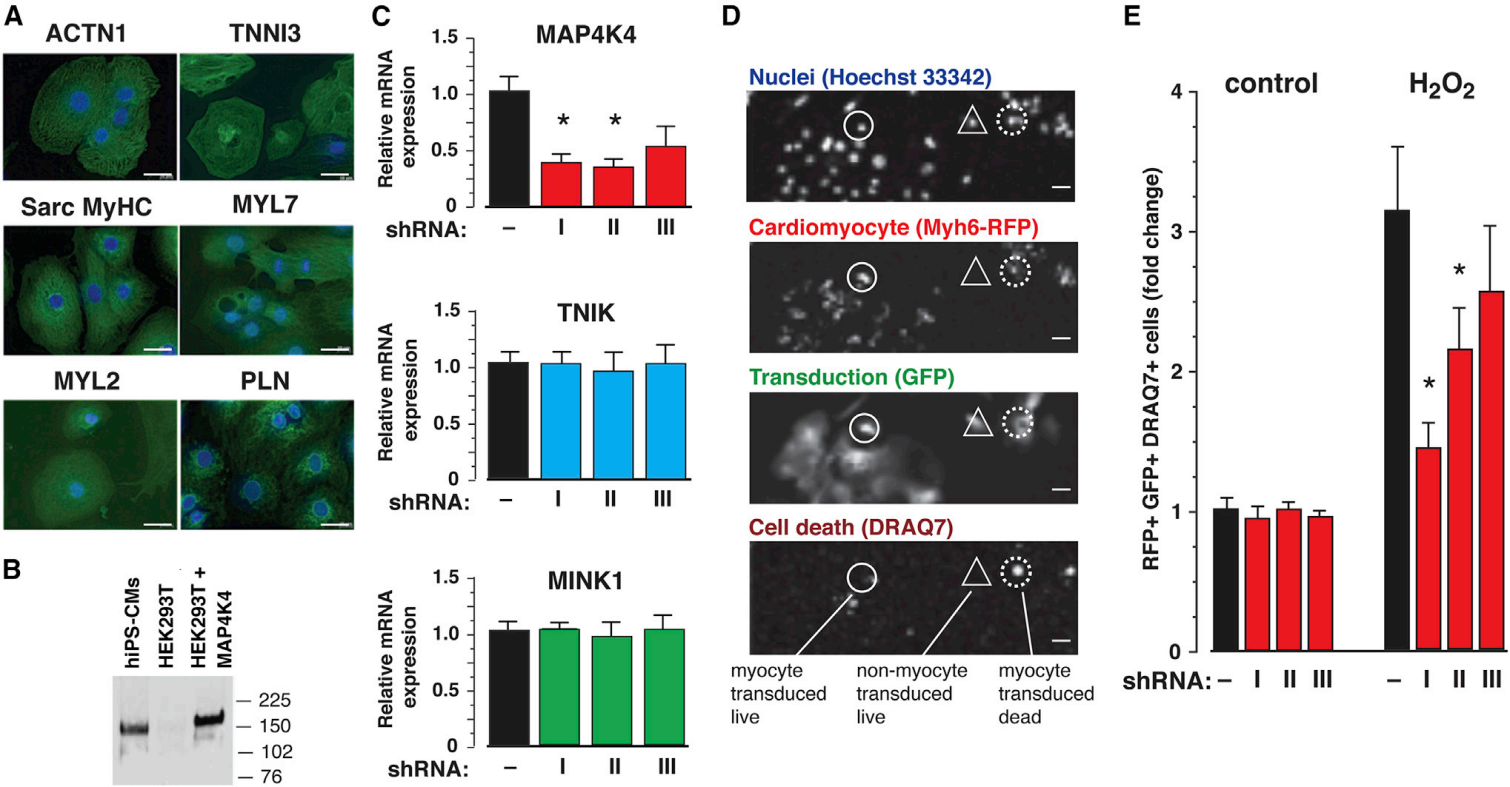
Target Validation for MAP4K4 by Gene Silencing in Human Stem Cell-Derived Cardiomyocytes (A) Prevalence of the indicated cardiomyocyte markers, by immunostaining, ranged from 80% to 90%. iCell cardiomyocytes were used here and in subsequent panels. Scale bars, 20 μm. (B) MAP4K4 protein expression in hiPSC-CMs by immunoprecipitation then western blotting. Untransfected and MAP4K4-transduced HEK293T cells are shown for comparison. (C) Potency of the *MAP4K4* shRNAs in hiPSC-CMs by qPCR. n = 2 independent experiments, 3 replicates in each; ^∗^p ≤ 0.05. Specificity of the *MAP4K4* shRNAs is shown by lack of effect on the two closest MAP4Ks, *TNIK* and *MINK*. I, II, and III in (C) and (E) denote V2LHS 68219, V2LHS 202781, and V2LHS 201856. (D and E) Protection of hiPSC-CMs from 250 μM H_2_O_2_ assessed as DRAQ7 uptake in successfully transduced (GFP^+^) cardiomyocytes (*Myh6*-RFP^+^). (D) Representative images from the high-content assay for the channels shown. Phenotypes are highlighted for three complementary indicative cells. Circle, myocyte; triangle, non-myocyte; solid line, live; dotted line, dead. Scale bars, 20 μm. (E) Protection by the two potent shRNAs shown in (C). n = 2 independent experiments, 6 replicates in each; ^∗^p ≤ 0.05. Results in (C) and (E) are shown as the mean ± SE.

First, the prevalence of cardiomyocyte-specific markers and presence of MAP4K4 protein were validated in iCell cardiomyocytes (Ma et al., 2011; Figures 1A and 1B). Two of three shRNAs directed against human *MAP4K4* reduced expression >60%, with no extraneous effect on *MINK1/MAP4K6* and *TNIK/MAP4K7*, the most closely related genes (Figure 1C). Cell death was quantified by high-content analysis as the loss of membrane integrity (DRAQ7 uptake) in successfully transduced (GFP^+^) hiPSC-CMs (Myh6-RFP^+^; Figure 1D). In this four-channel assay, roughly 85% of cells were RFP^+^, half were GFP^+^ as well, and death was induced in half the control myocytes. Hence, the specifically relevant cells in which cardiomyocyte death occurred were a large sub-population, comprising roughly 20% of the culture. Each of the two most potent shRNAs conferred protection against H_2_O_2_: cardiomyocyte loss was reduced up to 50% (Figure 1E). By contrast, shRNA with little effect on MAP4K4 did not confer protection. Thus, the results of gene silencing strongly suggest a requirement for endogenous MAP4K4 in human cardiac muscle cell death.

### Pharmacological Inhibition of MAP4K4 Suppresses Human Cardiac Muscle Cell Death

**Figure 2:**
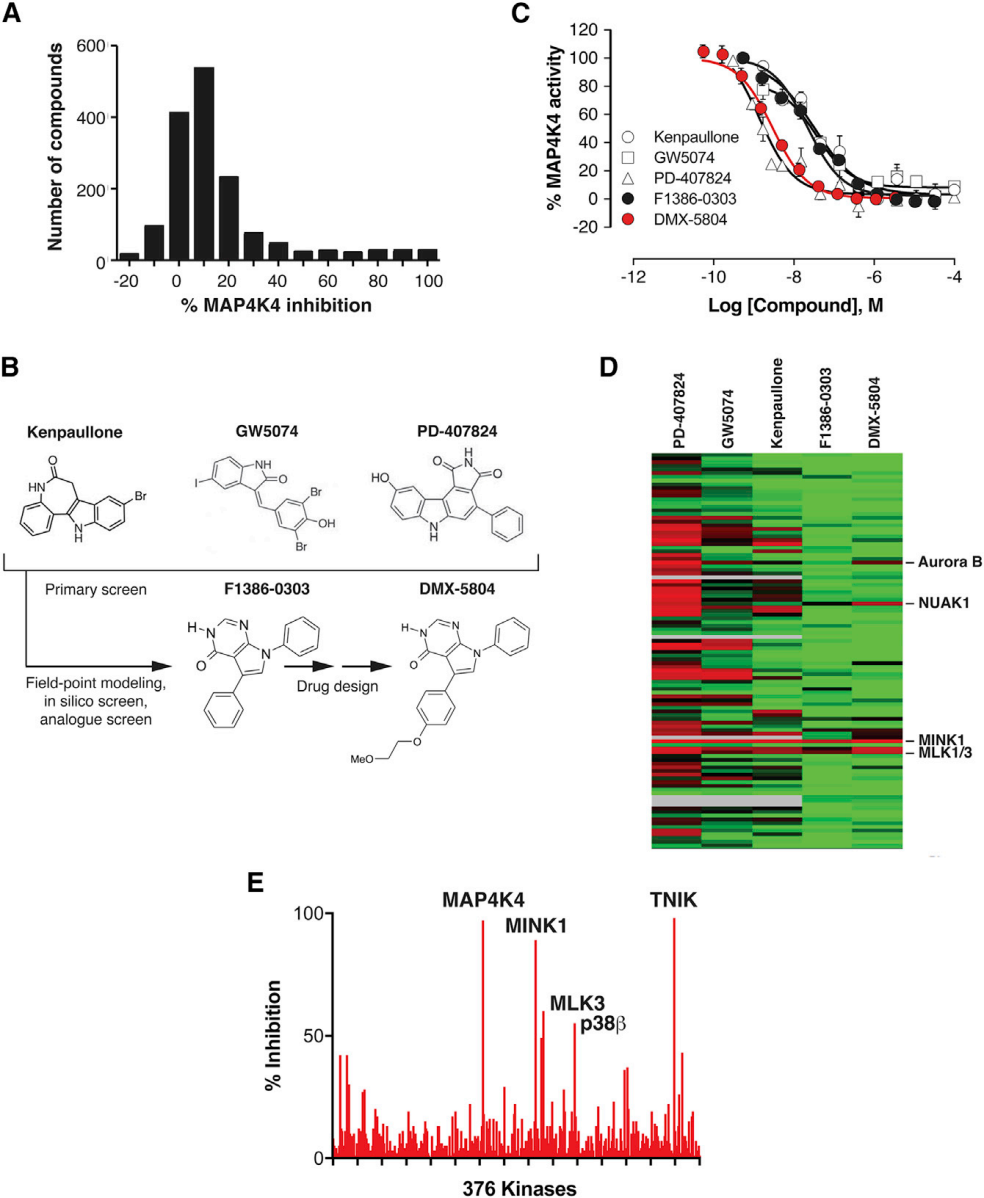
Selective Small-Molecule Inhibitors of MAP4K4 Created by Field-Point Modeling and Screening *In Silico* (A) Distribution histogram, showing the primary cell-free screen for inhibitors of human MAP4K4. (B) Chemical structures of top compounds from the primary screen, used for the pharmacophore template (Kenpaullone; GW5074; PD-407824), subsequent virtual screen (F1386-0303), and medicinal chemistry refinement (DMX-5804). (C) Dose-response relations for the compounds in (B), against recombinant human MAP4K4. IC_50_ values were as follows: Kenpaullone, 30 nM; GW5074, 30 nM; PD-407824, 1.5 nM; F1386-030, 34 nM; and DMX-5804, 3 nM. (D) Selectivity profile at 1 μM, shown as a heatmap of residual activity for 141 human kinases in the presence of the indicated compounds. Highlighted at the right for comparison are the few off-target kinases affected by DMX-5804 at this concentration (≤30% activity). Red, 0%; black, 50%; green, 100%; gray, not tested. (E) More extensive selectivity profile of DMX-5804 (376 kinases), at 30× its IC_50_ against MAP4K4. Results are shown as the mean ± SE.

Based on this portfolio of results—ranging from human to rodent and back—we postulated that suppressing MAP4K4 pharmacologically would reproduce the benefit achieved by gene silencing and, consequently, promote human cardiac muscle cell survival. Therefore, we sought to identify novel small-molecule inhibitors with sufficient potency and selectivity for further development toward clinical application. A three-step approach was undertaken: (1) empirical screening against human MAP4K4, using ∼1,800 biologically active compounds (Figure 2A); (2) molecular field-based similarity analysis of the top primary hits, using the consensus pharmacophore as seed for a ligand-based virtual screen (Cheeseright et al., 2011), followed by substructure and similarity searches (Figure 2B); and (3) refinement of the resulting tool compound, F1386-0303 (5,7-diphenyl-7H-pyrrolo[2,3-d]pyrimidin-4-ol), by rational drug design (Figure 2B). Chemical structures are shown in Figure 2B and full dose-response data in Figure 2C. Compared to the promiscuity of primary hits, F1386-0303 was far more selective (25–70 off-target effects reduced to 5, among 141 human kinases: Figure 2D and Table S2), with equivalent potency directed solely against the closest relatives, MINK1 and TNIK (Table 1).

**Table 1:** Comparison of DMX-5804 and the Starting Compound F1386-0303

Selectivity			
**Target**	**F1386-0303 pIC50 (Fold Selectivity)**	**DMX-5804 pIC50 (Fold Selectivity)**	**DMX-5804 vs F1386-0303**

MAP4K4	7.46	8.55	–
MINK1/MAP4K6	7.42	8.18	–
TNIK/MAP4K7	7.03	7.96	–
GCK/MAP4K2	5.91 (35)	6.50 (112)	3.20
GLK/MAP4K3	4.52 (871)	4.95 (3981)	4.57
KHS/MAP4K5	5.22 (174)	6.36 (153)	0.88
ABL1	4.52 (865)	5.80 (560)	0.65
Aurora B	4.88 (380)	5.49 (560)	1.47
FLT3	5.66 (63)	5.31 (1148)	18.22
GSK3β	4.57 (776)	4.66 (7762)	10.00
MLK1/MAP3K9	6.28 (15)	7.19 (23)	1.53
MLK3/MAP3K11	6.09 (23)	6.99 (36)	1.57
NUAK	6.16 (20)	6.88 (47)	2.35
VEGFR	5.72 (55)	5.72 (675)	12.27
Pharmacokinetics			

**Target**	**F1386-0303**	**DMX-5804**	**DMX-5804 versus F1386-0303**

**IV PK (1 mg kg**^**−1**^**)**			

Cl (L hr^−1^ kg^−1^)	5.33	2.50	0.47
t_1/2_ (h)	0.1	0.6	6.00
C_max_ (nM)	3262	1590	0.49
V_d_ (L kg^−1^)	1.05	1.22	1.16

**Oral PK (50 mg kg**^**−1**^**)**			

AUC_inf_	2162	63733	29.48
C_max_ (nM)	295	13847	46.94
T_max_ (h)	1.00	1.00	1.00
t_1/2_ (h)	3.7	1.8	0.49

Selectivity: the top compound from pharmacophore modeling (F1386-0303) and its derivative DMX-5804 were tested for activity against MAP4K4 and selected other human protein kinases (HTRF Transcreener ADP assay). Apart from related MAP4Ks, the kinases tested for full dose-response relations were chosen on the basis of off-target activity in the Dundee selectivity panel (Table S2). VEGFR was detected as an off-target hit of DMX-5804 at the dose used for compound screening, but is nearly 700-fold less sensitive than MAP4K4. Pharmacokinetics: plasma concentrations were determined after intravenous or oral administration at the doses shown. AUC_inf_, area under the plasma concentration-time curve from time 0 to infinite; Cl, clearance; C_max_, peak concentration; t_1/2_, plasma half-life; T_max_, time of peak concentration; V_d_, volume of distribution.

To assess whether the compound traverses the cell membrane and inhibits endogenous MAP4K4, activity in HEK293T cells was assessed 1 h after treatment at 10 μM. F1386-0303 inhibited endogenous MAP4K4 activity by 70% (Figure S4E). In rat ventricular myocytes, cell death induced by H_2_O_2_ was inhibited ∼50% at this concentration (Figure S4F), equaling the protection conferred by *Map4k4* shRNA (Figures S4C and S4D). No adverse effect of F1386-0303 was detected at any of the concentrations, and rat cardiomyocyte death was inhibited by the compound even at 1 μM.

**Figure 3:**
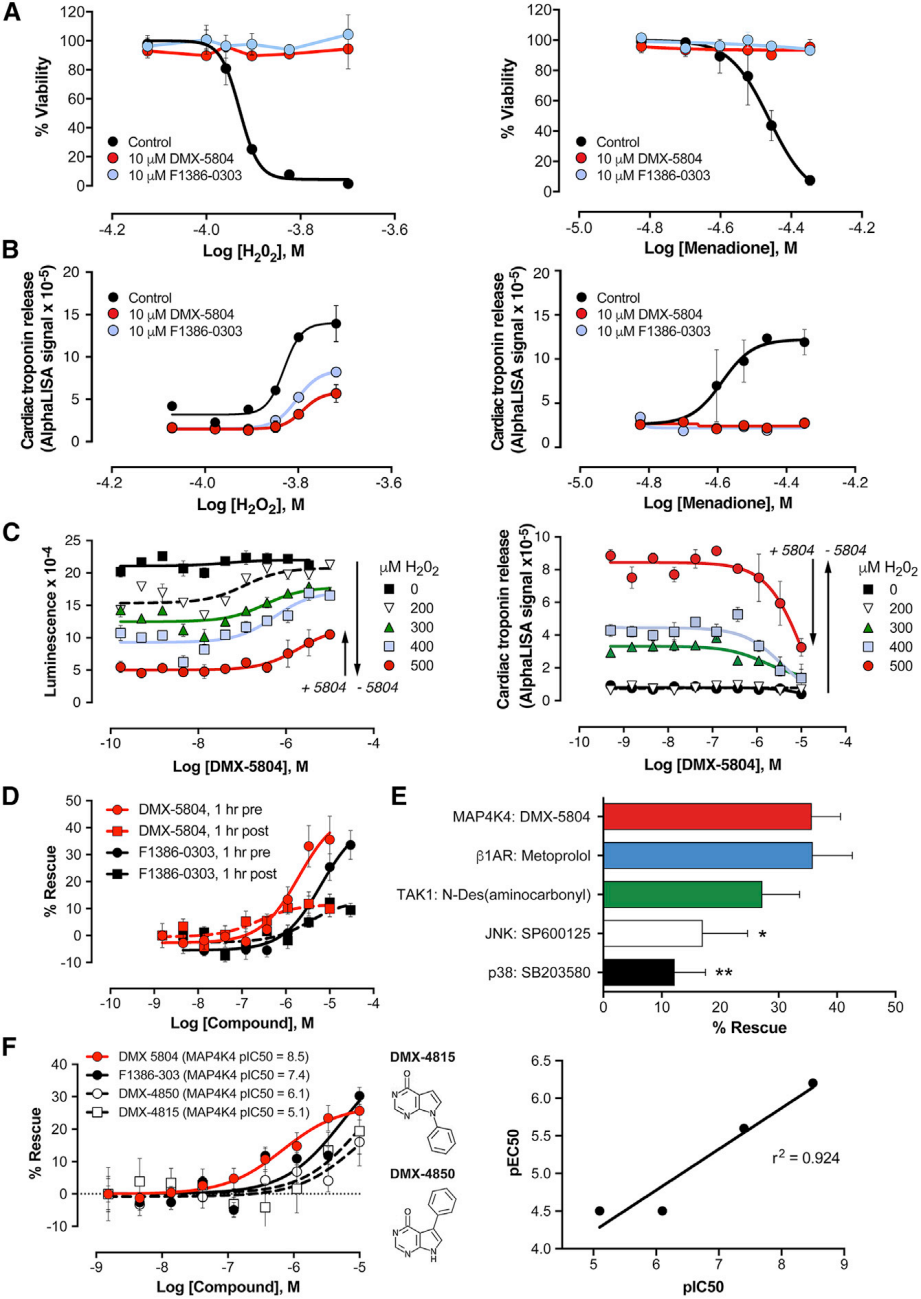
Protection of Human Stem Cell-Derived Ventricular Myocytes by Small-Molecule Inhibitors of MAP4K4 (A and B) vCor.4U ventricular myocytes were assayed 24 h after oxidative stress conferred by H_2_O_2_ (left) or menadione (right) at the indicated concentrations, ±DMX-5804 or the parent compound F1386-0303 (10 μM) 1 h prior to insult. Cardiomyocyte protection was confirmed in three independent experiments, using three different lots of vCor.4U cells (CV98CL V, CV99CL V, and CV102CL V). A representative dose-response curve is shown in each panel (2 replicate wells per condition). (A) CellTiter-Glo assay. Results (% viability) are normalized to the difference between untreated control cells (no death signal and no inhibitor) versus 100% cell death (0.1% Triton X-100 2 h before CellTiter-Glo [CTG]). (B) Human cardiac troponin I release (AlphaLISA). (C) Cross-titration of DMX-5804 and H_2_O_2_. (Left) CellTiter Glo is shown; (right) troponin release is shown; n = 10. Arrows illustrate the loss of viability at 500 μM H_2_O_2_ and rescue by 10 μM DMX-5804. The half-maximal concentration for protection shifts systematically rightward as oxidative stress increases. To minimize inter-experimental variation, results at a fixed concentration of death signal (D–F: CellTiter Glo; % rescue) are normalized to the difference between untreated control cells versus stress-induced death in the absence of inhibitor. (D) Partial protection by MAP4K4 inhibitors given 1 h before versus 1 h after 400 μM H_2_O_2_; N ≥ 3. (E) vCor.4U cells were stressed with 400 μM H_2_O_2_ 1 h after treatment with the compounds shown. Nine-point dose-response curves were obtained; for clarity, rescue of viability is shown as a bar graph at the highest concentration (10 μM). Fidelity to pathways driving infarct size in human trials is suggested by the positive result for β1-adrenergic blockade and weak result for p38. Inhibition of the MAP4K4 target TAK1 was likewise effective; N ≥ 3; ^∗^p = 0.0225; ^∗∗^p = 0.0011. (F) (Left) Dose-response relations for in-cell activity, measured as protection from 400 μM H_2_O_2_ (CellTiter-Glo). (Right) Potency for human cardiomyocyte protection correlates with the potency against recombinant human MAP4K4, across three orders of magnitude. Results are shown as the mean ± SE.

To substantiate these benefits in a human milieu, cytoprotection was next assessed using hiPSC-CMs. Pharmacological inhibition by F1386-0303 was highly protective, suppressing human cardiac muscle cell death in iCell cardiomyocytes even at micromolar concentrations (DRAQ7 uptake; Figure S5A), equaling the benefit achieved by gene silencing. Human cardiac muscle cell protection was substantiated in a second line, vCor.4U cardiomyocytes, which was likewise validated as physiologically predictive (Blinova et al., 2018, El-Haou et al., 2018) but is more highly enriched for ventricular myocytes—the clinically relevant subtype. The ventricle-specific protein MLC_2V_ was readily detected in >80% of vCor.4U cardiomyocytes (Figure S5B) but just a minority of iCell cardiomyocytes (Kattman et al., 2011). As complementary forms of oxidative stress, vCor.4U cells were treated with increasing concentrations of H_2_O_2_ or of menadione (vitamin K3), which induces intracellular reactive oxygen species through quinone redox cycling (Badave et al., 2016). At 10 μM F1386-0303, protection from either death signal was virtually complete (CellTiter-Glo, Figure 3A; human cardiac troponin release, Figure 3B), even at the highest concentrations used (200 μM H_2_O_2_ or 45 μM menadione). Thus, F1386-0303 is a potent, selective MAP4K4 inhibitor that successfully protects human stem cell-derived ventricular cardiomyocytes from lethal oxidative stress.

### An Enhanced MAP4K4 Inhibitor Rescues Human Stem Cell-Derived Cardiomyocyte Viability and Function

F1386-0303 does not, however, have sufficient bioavailability in mice to be used for proof-of-concept studies *in vivo*: it is rapidly cleared and accumulates only to low levels when dosed orally in mice (Table 1). Analogues of F1386-0303 were therefore created to improve these properties. Notable among these was 5-[4-(2-methoxy-ethoxy)-phenyl]-7-phenyl-3,7-dihydro- pyrrolo[2,3-d]pyrimidin-4-one, designated DMX-5804 (Figure 2B), which was more potent (Figure 2C), selective (Figures 2D and 2E; Tables 1 and S2), stable *in vivo* (Table 1), and taken forward for systematic testing in the human cardiomyocytes.

First, protection was substantiated at 10 μM using vCor.4U cells as above (Figures 3A and 3B). Across a series of experiments with DMX-5804, the effective concentration that gives a half-maximal response (EC_50_) was 500 nM for cell death induced by 400 μM H_2_O_2_, though contingent on the level of stress (Figure 3C), time of administration (Figure 3D), and specific human cardiomyocyte line of the three tested (Figure S5C). Of translational significance, partial protection was seen, even if given 1 h after oxidative stress. Although these complementary lines differ in their human donor, tissue source, reprogramming vectors, pluripotency genes, methods of differentiation, and maintenance media (Burridge et al., 2014, Ma et al., 2011, Yu et al., 2007), all three showed the same level of maximal protection. In vCor.4U cells, this dose response was shifted toward higher concentrations of compound, which may reflect greater vulnerability of ventricular myocytes to this form of stress or merely technical differences across the lines.

Notably, human cardiomyocyte survival also was promoted in this model by a beta-adrenergic blocker, metoprolol, that reduces infarct size in humans (Ibanez et al., 2013), but not by inhibition of p38 (Figure 3E), a pathway that failed to show importance in human trials despite credible large-mammal evidence (Barancik et al., 2000, Newby et al., 2014). This dichotomy, although retrospective, suggests the predictive power of hiPSC-CMs, over standard animal models alone, in prioritizing targets for reducing human infarct size. Under these conditions, inhibition of the MAP4K4 target TAK1 (MAP3K7) likewise was effective.

Compared with F1386-0303, DMX-5804 showed 10-fold greater potency not only in suppressing recombinant MAP4K4 activity (inhibitor concentration that gives 50% inhibition [IC_50_], 3 versus 34 nM; Figure 2C) but also in protecting hiPSC-CMs (EC_50_ in iCell cardiomyocytes, 0.5 μM versus 5 μM; Figure 3F). This strict correspondence between cytoprotection and MAP4K4 inhibition was seen further using two other derivatives of F1386-0303, whose potency in both settings is diminished at least 10-fold (Figure 3F).

Protection of human cardiomyocyte survival by DMX-5804 also was confirmed using hypoxia-reoxygenation (Figure S5D), an alternative surrogate for ischemia-reperfusion *in vivo*. However, the hiPSC-CMs were less sensitive to hypoxia-reoxygenation than to other death signals tested, in agreement with resistance reported by others (Chen and Vunjak-Novakovic, 2018, Hidalgo et al., 2018).

**Figure 4:**
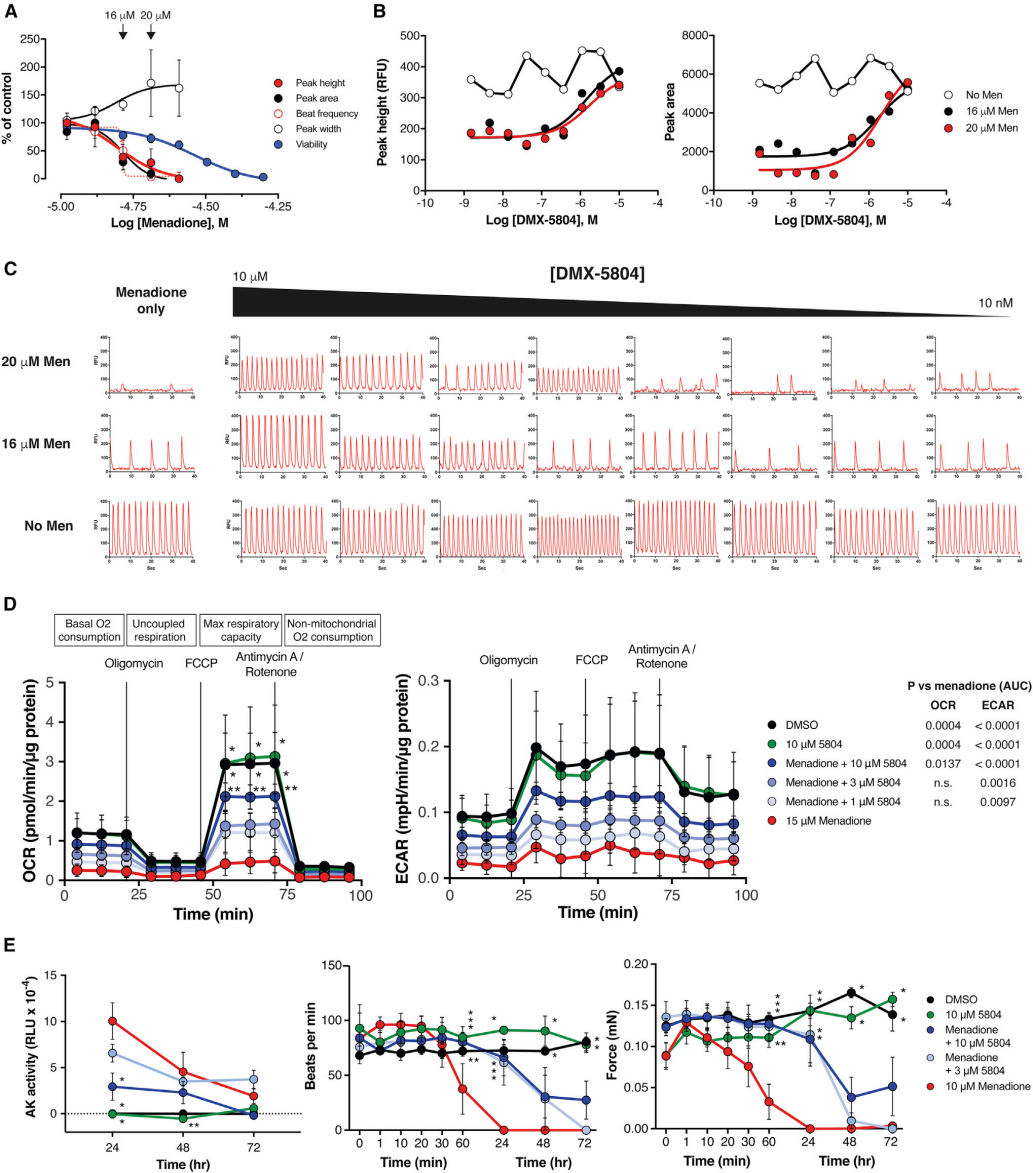
MAP4K4 Inhibition Rescues Mitochondrial Function, Calcium Cycling, and Contractile Function in Human Ventricular Myocytes (A–C) Calcium oscillations in vCor.4U cells. Protection was confirmed in three independent experiments; one representative set of dose-response curves is shown (4 replicate wells for each condition). (A) Spontaneous calcium transients at 24 h were suppressed by menadione even at sub-lethal concentrations (CellTiter-Glo assay). (B) Rescue by DMX-5804. (C) Representative calcium transients. (Left) DMX-5804 had no effect on baseline calcium cycling. (Right) Preservation of calcium cycling by DMX-5804 in menadione-treated cells is shown. (D) Mitochondrial function was assessed in vCor.4U cells using menadione as the oxidative stress (15 μM; 2 h), followed by the sequential inhibitors shown. Mitochondrial respiration (left) and glycolysis (center) were suppressed by menadione and rescued by 10 μM DMX-5804. For pairwise comparisons, ^∗^p < 0.001 versus menadione; ^∗∗^p ≤ 0.01 versus menadione. p values (right) denote treatment effects calculated as area under the curve (AUC). (E) vCor.4U cells were cultured as 3D engineered heart tissue and subjected to menadione for 24 h ± DMX-5804. (Left) Suppression of cell death at 24 h is shown. ^∗^p < 0.001 versus menadione; ^∗∗^p = 0.02 versus menadione. (Center) Preservation of spontaneous beating at 1 and 24 h is shown. ^∗^p < 0.001 versus menadione; ^∗∗^p ≤ 0.03 versus menadione. (Right) Preservation of force generation is shown. ^∗^p < 0.001 versus menadione; ^∗∗^p = 0.008 versus menadione. Results are shown as the mean ± SE.

Given its potency, selectivity, and cytoprotective effects, DMX-5804 next was investigated for its ability to preserve key aspects of human cardiomyocyte function after oxidative stress (Figure 4). Calcium cycling, a hallmark of the cardiac phenotype, is susceptible to redox- and phosphorylation-dependent abnormalities (Meissner, 2017). To determine whether MAP4K4 inhibition might preserve calcium homeostasis, vCor.4U hiPSC-CMs were assessed using menadione plus an intracellular calcium indicator (EarlyTox Cardiotoxicity Dye; Molecular Devices; Figures 4A–4C). Calcium cycling was sensitive to oxidative stress even at sub-lethal concentrations of menadione, with half-maximal loss of peak area and peak height at 16 μM, at which no confounding effect on cell viability is observed. The concentration at which DMX-5804 preserved ∼50% of peak area and peak height was 0.5–2 μM, and varied with the level of dysfunction conferred by menadione in each experiment. Protection was maintained for up to 96 h, despite the presence of menadione throughout. Similar to its greater effects both *in vitro* and in cell survival (Figures 2C and 3F), DMX-5804 was 5- to 10-fold more potent than F1386-0303 in protecting calcium cycling under the sub-lethal conditions tested (Figure S5E). Although the tested protocol for hypoxia-reoxygenation provoked less than 20% cell death, spontaneous calcium cycling was markedly inhibited, and protection was conferred by DMX-5804 (Figure S5F).

Maximum oxidative capacity, a measure of mitochondrial respiration, was reduced to 15% of control levels by menadione (15 μM; 2 h), and residual activity was improved 5-fold by 10 μM DMX-5804 (Figure 4D, left). No significant benefits in this parameter were seen at lesser concentrations. Likewise, 10 μM DMX-5804 partially rescued the extracellular acidification rate, a measure of glycolytic function (Figure 4D, right).

Next, protection was assessed in vCor.4U cells configured as 3D human-engineered heart tissue (hEHT), a model in which the maturity of physiological properties is further enhanced (Hansen et al., 2010, Lemoine et al., 2017, Weinberger et al., 2017). Cell death from menadione was greatest in the first 24 h after treatment and suppressed by 10 μM DMX-5804 (Figure 4E). Spontaneous beating and, hence, force generation were decreased by menadione within 60 min and were abolished at 24 h. The rescue of contractile function by 3 or 10 μM DMX-5804 was virtually complete at these time points, although not later ones.

Of relevance to potential future safety considerations, no adverse effect of DMX-5804 was seen in 2D culture or hEHT on any of the parameters tested—cardiomyocyte viability, rhythmicity, calcium handling, mitochondrial function, and force generation. Likewise, no off-target effect was seen in cloned human ion channels (hERG, hNaV1.5, and hCaV1.2: Figure S5G), Ames test of mutagenic potential, glutathione (GSH)-trapping, or Cerep Safety 44 panel, except metallothionein 3.

### MAP4K4 Inhibition Reduces Infarct Size in Mice

**Figure 5:**
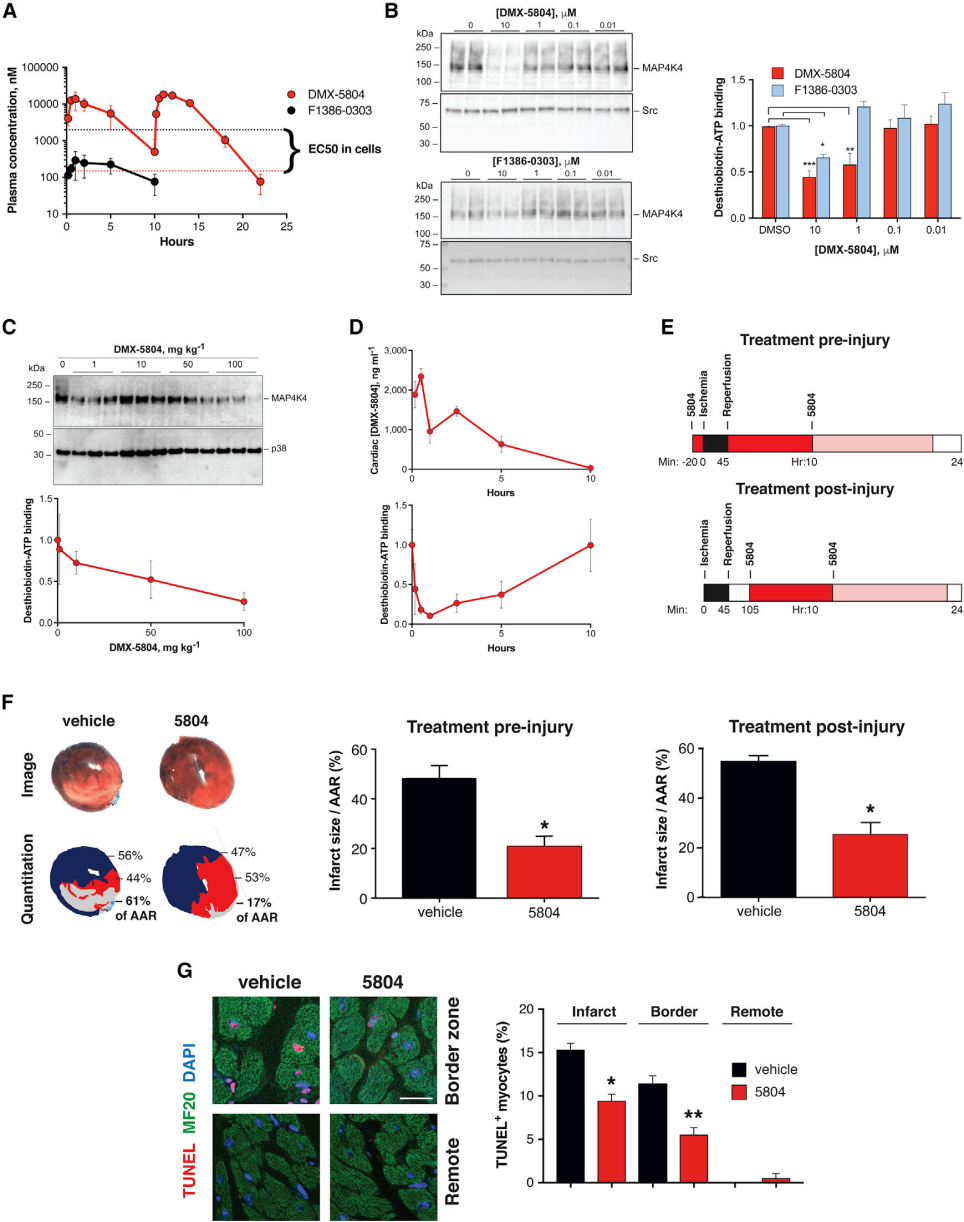
MAP4K4 Inhibition Reduces Infarct Size in Mice (A) Plasma concentrations of DMX-5804 versus F1386-0303 in mice, after oral doses of 50 mg kg^−1^. Whereas F1386-0303 levels were 10-fold less than the compound’s EC_50_ (black), sufficient *in vivo* coverage was achieved with DMX-5804 (red). Using a second dose at 10 h, levels exceeded the EC_50_ for nearly 1 day. (B–D) Target engagement, shown as a block to desthiobiotin-ATP binding by cardiac MAP4K4 ± the ATP-competitive inhibitors. (B) Target engagement *ex vivo*, in adult mouse cardiac lysates, demonstrates 10× greater affinity for DMX-5804. (C and D) Target engagement *in vivo*, shown for (C) dose response and (D) time course. Cardiac levels of DMX-5804 (above) conformed to the time course for plasma concentrations and reciprocal with the levels of ATP binding to cardiac MAP4K4 (below). (E) Schematic representation of the pre- and post-injury treatment protocols (not to scale). Mice were given the first dose of DMX-5804 20 min before the onset of ischemia (above) or 1 h after reperfusion was initiated (below). Black, ischemia; red and light red, intervals of coverage by the first and second dose, respectively. Dosing was chosen to exceed the compound’s EC_50_ for 20 h (Figure 2E). (F) Infarct size. (Left) Representative tissue sections 24 h after myocardial infarction ± DMX-5804 are shown. (Above) TTC/Evans blue staining to delineate infarct size (white) and area at risk (AAR) is shown. (Below) Image analysis and quantitation are shown. (Center) Pre-injury treatment with DMX-5804 reduces infarct size/AAR. N ≥ 5; ^∗^p = 0.0021. (Right) Post-injury treatment with DMX-5804 reduces infarct size/AAR. N ≥ 5; ^∗^p = 0.0015. (G) Cardiomyocyte apoptosis. (Left) Representative confocal microscopy of cardiomyocyte apoptosis 24 h after myocardial infarction ± DMX-5804 is shown. Myocytes are shown in short-axis orientation. Scale bar, 20 μm. (Right) Post-injury treatment with DMX-5804 reduces cardiomyocyte apoptosis. n = 4; ^∗^p = 0.0014; ^∗∗^p = 0.0028. Representative images (F and G) are taken from the post-injury treatment study. Results are shown as the mean ± SE.

To test whether MAP4K4 target validation and compound development in hiPSC-CMs might predict success in a whole-animal context, mice undergoing experimental myocardial infarction were treated with DMX-5804 or the vehicle control (Figure 5). As a result of its reduced clearance, the free plasma concentrations of DMX-5804 were 334 and 8 nM, respectively, 1 and 10 h after a 50 mg kg^−1^ oral dose, showing more than 80-fold improvement over the initial inhibitor tested (Figure 5A; Table S4). Target engagement measured *ex vivo*, as a block to desthiobiotin-ATP binding (Patricelli et al., 2011), demonstrated 10× greater affinity for DMX-5804 over the earlier compound in adult mouse cardiac lysates (Figure 5B). To provide direct proof of target engagement *in vivo*, cardiac lysates after oral dosing were incubated with the covalent ATP probe, and MAP4K4 was immunoprecipitated for dose-response and time course studies (Figures 5C and 5D). The inhibition of ATP binding to cardiac MAP4K4 was substantiated for DMX-5804 given *in vivo* and corresponded well to tissue levels of the compound. Based on these pharmacokinetic and pharmacodynamic results, mice received 50 mg kg^−1^ twice by gavage, spaced 10 h apart, to achieve coverage for nearly a day exceeding the measured EC_50_ for cardiomyocyte protection (Figure 5E). Treatment was begun either 20 min prior to ischemia or 1 h after reperfusion injury, the latter having greater relevance to potential clinical benefits. Suppression of total cardiac muscle cell death by more than half was achieved in both studies, reducing infarct size as a proportion of the area at ischemic risk (pre-injury: 48.5% versus 20.9%; post-injury: 55.1% versus 25.6%; Figure 5F). As an independent and selective measurement of cell death, TUNEL staining was performed in the post-injury study, demonstrating suppression of cardiomyocyte apoptosis within the infarct itself and adjacent jeopardized myocardium by 39% and 52%, respectively (Figure 5G). Given the known large contributions of both apoptosis and necrosis to infarct size (Whelan et al., 2010), the net effect we demonstrated cannot be reconciled with just a partial block to apoptosis alone.

## Discussion

A scientific case for MAP4K4 is suggested by many conventional criteria (“observational” biochemistry, mouse models, and rodent cardiomyocytes), although these, even taken together, are merely tentative or provisional indications of a functional role in humans. This standard pipeline for cardiac drug discovery—“flying blind” with respect to human efficacy—is notoriously failure prone (Fordyce et al., 2015, Gromo et al., 2014), prompting academic and industry-based investigators alike to embrace target validation in human cardiomyocytes from pluripotent stem cells as an accessible, scalable, transformative alternative (Bellin et al., 2012, Burridge et al., 2016, Cameron et al., 2013, Gintant et al., 2017, Lee et al., 2017b, Liang et al., 2013, Mathur et al., 2015, Matsa et al., 2014, Matsa et al., 2016, Sharma et al., 2017). Here, using hiPSC-CMs as the most relevant platform for gene silencing and drug discovery, we created small-molecule inhibitors of MAP4K4 through field-point modeling and screening *in silico*. F1386-0303 mimics the protective effect of MAP4K4 shRNA in the human cardiomyocytes. A further drug discovery endeavor led from this initial tool compound to one suitable for *in vivo* studies, DMX-5804, enhanced ten-fold for potency both against the recombinant human kinase and against cell death in human cardiac muscle cells. Whereas the earlier compound was unsuited to whole-animal testing on the grounds of rapid clearance and poor bioavailability, DMX-5804 successfully reduced infarct size in mice, even given 1 h after reperfusion injury. The solubility and pharmacokinetic properties of DMX-5804 itself remain insufficient for a human drug candidate in acute ischemic injury, where rapid intravenous infusion is desired. However, this chemical series yielded useful tool compounds that enabled us to conduct rigorous validation in human cardiomyocytes, taken forward successfully into proof-of-concept studies *in vivo*. On this basis, we propose that pharmacological inhibition of MAP4K4 is a conceptually sound target for further development toward rational cardioprotection in the clinic, taking intravenous analogs forward into the requisite studies of toxicity, long-term efficacy, and large-mammal effects.

An agent that suppresses human cardiac muscle cell death is expected to provide additional benefit beyond current therapies, and our data suggest that inhibiting MAP4K4 might be a worthwhile approach to accomplish this. H_2_O_2_- or menadione-induced oxidative stress—the trigger of cell death tested here—replicates key features of both acute and chronic heart disease (Brown and Griendling, 2015). Though the molecular events driving MAP4K4 activation by oxidative stress are not yet known, the *a priori* possibilities include receptor-mediated signals, activation of an upstream signaling protein like RAP2 (Meng et al., 2018) or PYK (Loftus et al., 2013), or direct biochemical activation by caspase cleavage (Chang et al., 2006) or redox sensing (Burgoyne et al., 2007, Guo et al., 2010, Nadeau et al., 2009). Interestingly, effectors of MAP4K4 have been proposed as therapeutic targets in cardiovascular disease and neuronal injury: at least in preclinical studies, inhibiting TAK1/MAP3K7 is protective in ischemic stroke (White et al., 2012). Moreover, blocking MAP4K4 along with TNIK and MINK (our principal off-target effects) is beneficial in neurodegeneration (Larhammar et al., 2017).

Numerous logical targets to enhance cardiac muscle cell survival have failed in human studies, but all thus far, without exception, were taken forward on the basis of non-human models alone (Hausenloy and Yellon, 2015, Heusch, 2013, Lincoff et al., 2014, Newby et al., 2014, Piot et al., 2008). Of even more general importance, therefore, our studies reinforce the utility of human stem cell-derived cardiomyocytes, not solely for safety pharmacology and patient-specific models of inherited cardiac disorders, but also toward target discovery and drug development in acute myocardial infarction, a pandemic form of acquired heart disease. Suggesting the fidelity and potential predictive power of this model, the extent of protection conferred by inhibiting MAP4K4 was equal to that achieved with beta_1_-adrenergic blockade (one of the very few pathways that succeeded in the clinic) and far surpassed that with inhibition of p38 (a pathway that did not; Ibanez et al., 2013, Newby et al., 2014). We suggest this dichotomy lends credence to the cells’ use to help prioritize among the myriad of possible targets in cardiac muscle cell death, acting in series or in parallel with MAP4K4.

Moreover, beyond its central and indispensable role toward helping ensure target validity, our use of human stem cell derivatives provided immediate evidence of safety in the human cardiac context (no cytotoxicity, arrhythmia, disturbance of calcium cycling, or impaired force generation), as well as pinpointing the required concentrations to inform human dosing. It will be intriguing in future studies to determine which exact substrates and pathways are the responsible mediators of MAP4K4 in human cardiomyocytes, to define the mechanism(s) of MAP4K4 activation, and ultimately to identify the acute and chronic human cardiac disorders in which MAP4K4 inhibition might be most beneficial. An acknowledged limitation of hiPSC-CMs and other reductionist systems by comparison to the native adult organ is incomplete recapitulation of *in vivo* biology, attributable to functional immaturity and the lack of key cell-cell interactions. Offsetting this admitted disadvantage, even 2D cultures of pure hiPSC-CMs have well-demonstrated predictive power (Burridge et al., 2016, Devalla et al., 2015, Gintant et al., 2017, Matsa et al., 2014, Matsa et al., 2016), with even higher fidelity attained in the 3D human-engineered heart tissue we used (Lemoine et al., 2017). Refinements suitable for future work include enhancing metabolic maturation (Chen and Vunjak-Novakovic, 2018, Hidalgo et al., 2018) and heart-on-chip models that incorporate multiple cell types (Lemoine et al., 2017, Ronaldson-Bouchard et al., 2018). More than 30 mouse models exist that implicate specific protein kinases in cardiomyocyte death or survival, with no human proof of function to date (Fiedler et al., 2014). How might one prioritize among these, toward more effective cardiac drug discovery? By analogy to the instrumental role in drug discovery for human cancer cell lines (Wilding and Bodmer, 2014), we speculate that therapeutic targets and interventions that hold true not merely in animal models but also in human cardiomyocytes and 3D heart tissue from pluripotent stem cells may be prudent, well-posed choices for advancement toward human trials of suppressing cardiac muscle cell death.

## STAR★Methods

### Key Resources Table

**Table:** 

REAGENT or RESOURCE	SOURCE	IDENTIFIER
**Antibodies**		

Mouse monoclonal anti-ACTN2	Sigma-Aldrich	EA-53: A7811; RRID:AB_476766
Mouse monoclonal anti-FLAG epitope	Sigma-Aldrich	M2; RRID:AB_262044
Mouse monoclonal anti-HA epitope	Santa Cruz	F-7; RRID:AB_627809
Mouse monoclonal anti-MAP4K4	Abcam (Figure 1); CST (Figure 5)	ab56569; RRID:AB_944156 (Figure 1); 3485S; RRID:AB_2140972 (Figure 5)
Mouse monoclonal anti-MYH6	R&D Systems	MAB8979
Mouse monoclonal anti-MYL2	Synaptic Systerms	310 111; RRID:AB_887738
Mouse monoclonal anti-MYL7	Synaptic Systerms	311 011; RRID:AB_887737
Mouse monoclonal anti-PLN	Enzo Life Sciences	2D12: ALX-804-093-R100; RRID:AB_2052228
Mouse monoclonal anti-sarcomeric MyHC	R&D Systems	MF20: MAB4470; RRID:AB_1293549
Mouse monoclonal anti-sarcomeric MyHC, fluorescein-conjugated	R&D Systems	MF20: IC4470F; RRID:AB_1857205
Mouse monoclonal anti-sarcomeric tropomyosin	Sigma-Aldrich	T9283; RRID:AB_261817
Mouse monoclonal anti-Src	CST	2110S; RRID:AB_10691385
Mouse monoclonal anti-TNNT2	Santa Cruz	sc-52284; RRID:AB_630390
Mouse monoclonal IgG2b, fluorescein-conjugated	R&D Systems	Clone 133303: IC0041F; RRID:AB_357248
Goat anti-TNNI3	Santa Cruz	C-19; RRID:AB_793464
Goat anti-total actin (I-19), HRP-conjugated	Santa Cruz	I-19 (discontinued)
Goat anti-rabbit IgG, horseradish peroxidase-conjugated	Dako	P0448; RRID:AB_2617138
Rabbit anti-MAP4K4	This paper, method of Yao et al., 1999	https://www.ncbi.nlm.nih.gov/pubmed/9890973
Rabbit anti-ERK	Cell Signaling	9102; RRID:AB_330744
Rabbit anti-phospho-ERK (Thr202/Tyr204)	Cell Signaling	9101; RRID:AB_331646
Rabbit anti-JNK	Cell Signaling	9252; RRID:AB_2250373
Rabbit anti-phospho-JNK (Thr183/Tyr185)	Cell Signaling	9251; RRID:AB_331659
Rabbit anti-p38	Cell Signaling	9212; RRID:AB_330713
Rabbit anti-phospho-p38 (Thr180/Tyr182)	Cell Signaling	9211; RRID:AB_331641
Rabbit anti-caspase-3	Santa Cruz	H-277: sc-7148; RRID:AB_637828
Donkey F(ab’)_2_ fragment anti-mouse IgG (H+L), Alexa Fluor 488-conjugated	Jackson ImmunoResearch	715-546-150; RRID:AB_2340849
Donkey F(ab’)_2_ fragment anti-mouse IgG (H+L), Alexa Fluor 647-conjugated	Jackson ImmunoResearch	715-606-150; RRID:AB_2340865
Donkey anti-mouse IgG (H+L), Alexa Fluor 555-conjugated	Molecular Probes	A-31570; RRID:AB_2536180
Donkey F(ab’)_2_ fragment anti-rabbit IgG (H+L), Alexa Fluor 488-conjugated	Jackson ImmunoResearch	711-546-152; RRID:AB_2340619
Donkey F(ab’)_2_ fragment anti-rabbit IgG (H+L), Alexa Fluor 647-conjugated	Jackson ImmunoResearch	711-606-152; RRID:AB_2340625
Donkey anti-goat IgG (H+L), DyLight 649-conjugated	Jackson ImmunoResearch	705-606-147; RRID:AB_2340438

**Bacterial and Virus Strains**		

Lentivirus: GIPZ non-silencing control shRNAmir	Open Biosystems	RHS4348
Lentivirus: GIPZ MAP4K4 shRNAmir 68219	This paper	N/A
Lentivirus: GIPZ MAP4K4 shRNAmir 202781	This paper	N/A
Lentivirus: GIPZ MAP4K4 shRNAmir 201856	This paper	N/A
Adenovirus: MAP4K4	This paper	N/A
Adenovirus: MAP4K4 K54E	This paper	N/A
Adenovirus: MAP4K4 K54R	This paper	N/A
Adenovirus: JNK1 APF	This paper	N/A
Adenovirus: MAP4K4 shRNA	This paper	N/A
Adenovirus: GFP shRNA	This paper	N/A
Adenovirus: TAK1 K63W	Michael Schneider	https://www.ncbi.nlm.nih.gov/pubmed/10802712
Adenovirus: p38a AGF	Yibin Wang	https://www.ncbi.nlm.nih.gov/pubmed/9442057
Adenovirus: Bcl2	Lorrie Kirshenbaum	https://www.ncbi.nlm.nih.gov/pubmed/9315550
Adenovirus: LacZ	Michael Schneider	https://www.ncbi.nlm.nih.gov/pubmed/8326005

**Biological Samples**		

Human heart tissue	Sydney Human Heart Tissue Bank	HREC #2012/2814
Human heart tissue	DeBakey Heart Center, Baylor College of Medicine	H-15240

**Chemicals, Peptides, and Recombinant Proteins**		

Agarose	Invitrogen	15510-027
Antimycin A	Sigma-Aldrich	CAS 642-15-9
Aprotinin	Sigma-Aldrich	A1153
Captisol (sulfobutylether_7_-β-cyclodextrin)	Ligand Pharmaceuticals	https://www.ligand.com/technologies/captisol
Chemical libraries (virtual screen hits)	Enamine Screening Collection Chembridge Express Pick	https://enamine.net https://www.chembridge.com
Chemical library (primary screen)	ICCB Known Bioactives Library Tocriscreen Biologically Active Compound Library	Biomol BML-2840-0100 Tocris
Cor.4U Culture Medium	Axiogenesis	Ax-M-HC250
DePsipher (5,5′6,6’-tetrachloro-1,1’,3,3′-tetraethyl-benzimidazolylcarbocyanine iodide)	R&D Systems	CAS 3520-43-2
N-Des(aminocarbonyl)	Abcam	CAS 494772-87-1
DMEM	Biochrom	F0415
DMEM, 10x	GIBCO	52100-021
DMEM, high glucose, GlutaMAX supplement	Thermo Fisher Scientific	61965-026
DMEM, low glucose, GlutaMAX supplement, pyruvate	Thermo Fisher Scientific	10567-014
DMX-5804	This paper	PubChem accession number 98666
Doxorubicin	Calbiochem	CAS 23214-92-8
DRAQ7	Biostatus	CAS 1533453-55-2
Dulbecco’s Modified Eagle’s Medium (DMEM) Base, without glucose, L-glutamine, phenol red, sodium pyruvate and sodium bicarbonate	Sigma-Aldrich	D5030
F1386-0303	Domainex	CAS 287177-12-2
FCCP (carbonyl cyanide-4-phenylhydrazone)	Abcam	CAS 370-86-5
Fibrinogen	Sigma-Aldrich	F8630
Fibronectin, bovine plasma	Sigma-Aldrich	F1141
GNE-495	Domainex	CAS 1449277-10-4; https://www.ncbi.nlm.nih.gov/pubmed/26288693
Hoescht 33342	Molecular Probes	CAS 23491-52-3
Horse serum	GIBCO	26050
Human MAP4K4 kinase domain (aa 1-328)	Invitrogen	PV3687
HyClone defined fetal bovine serum	GE Healthcare Life Science	SH30070.03
iCell Cardiomyocytes Maintenance Medium	Cellular Dynamics	CMC-100-010-001
iCell Cardiomyocytes Plating Medium	Cellular Dynamics	CMC-100-010-001
Insulin	Sigma-Aldrich	I9278
L-alanyl-L-glutamine dipeptide (GlutaMAX-1)	Thermo Fisher Scientific	35050-061
L-Glutamine (200 mM)	Thermo Fisher Scientific	25030-081
Menadione	Sigma-Aldrich	CAS 58-27-5
Metoprolol (Succinate or tartrate)	Sigma-Aldrich	CAS 56392-17-7
Nec-1 s (7-Cl^−^O-*Nec*-*1*)	Merck	CAS 852391-15-2
Oligomycin A	Abcam	CAS 1404-19-9
Penicillin/streptomycin (10,000 U mL^−1^)	GIBCO	15140
Rotenone	Abcam	CAS 83-79-4
Rotenone	Abcam	CAS 83-79-4
SB203580	Sigma-Aldrich	CAS 152121-47-6
Sodium pyruvate (100 mM)	Thermo Fisher Scientific	11360-070
SP600125	Sigma-Aldrich	CAS 129-56-6
Texas Red-X succinimidyl ester	Molecular Probes	F-6162

**Critical Commercial Assays**		

Ames genotoxicity reverse mutation assay	Cyprotex	https://www.cyprotex.com/toxicology/genotoxicity/amestest
ApopTag Plus Fluorescein *In Situ* Apoptosis Detection Kit	Millipore	S7111
ArrayScan VTI High Content Screening platform	Cellomics	N/A
Cardiovascular ADR	Eurofins Cerep	N/A
CellTiter-Glo	Promega	G7570
CellTox Green	Promega	G8741
CLARIOstar with Atmospheric Control Unit	BMG Labtech	N/A
EHT Contraction Analyzer	EHT Technologies GmbH	A0001
EHT silicone racks	EHT Technologies GmbH	C0001
EHT Teflon spacers	EHT Technologies GmbH	C0002
FLIPR Tetra	Molecular Devices	N/A
HTRF Transcreener ADP	CisBio Bioassays	N/A
Human cardiac troponin I AlphaLISA	Perkin Elmer	AL295C
Ion channel safety screening	Patchliner (Nanion Technologies), Apconix	https://www.apconix.com/ion-channel-screening/
Kinase enrichment kit	Thermo Fisher Scientific	88310
Kinase inhibitor selectivity profiling	International Centre for Kinase Profiling, MRC Protein Phosphorylation and Ubiquitylation Unit	Premier Screen, http://www.kinase-screen.mrc.ac.uk
Kinase inhibitor selectivity profiling	Reaction Biology	www.reactionbiology.com/webapps/site/KinaseDetail.aspx
Microsome stability	Cyprotex	N/A
MitoXpress Intra Intracellular Oxygen Assay	Agilent	MX-300-4
Pharmacokinetic profiling	Pharmidex	N/A
PHERAstar Plus	BMG Labtech	N/A
SafetyScreen44	Eurofins Cerep	P270
Seahorse XFe24 Analyzer	Agilent	N/A
Seahorse XF24 FluxPak	Agilent	100850-001

**Deposited Data**		

DMX-5804 structure	PubChem	DMX-5804; accession number 98666

**Experimental Models: Cell Lines**		

Human embryonic kidney cells: HEK293T, female	Clontech	632180
Human iPSC-derived cardiomyocytes: vCor.4U, female	Ncardia (Axiogenesis)	Ax-B-HC03-1M
Human iPSC-derived cardiomyocytes: iCell, female	Cellular Dynamics	CMC-100-110-001
Human iPSC-derived cardiomyocytes; IMR-90, female	Sian Harding	https://www.ncbi.nlm.nih.gov/pubmed/29086457

**Experimental Models: Organisms/Strains**		

Mouse: FVB/N Tg(Myh6-Map4k4)	This paper	N/A
Mouse: FVB/N Tg(Myh6-Gnaq)25Gwd	Gerald Dorn	https://www.ncbi.nlm.nih.gov/pubmed/9576430
Mouse: FVB/N Tg(Myh6-Tnfa)	Douglas Mann	https://www.ncbi.nlm.nih.gov/pubmed/11502710

**Oligonucleotides**		

GIPZ shRNAmirs: human MAP4K4	Open Biosystems	V2LHS 68219, V2LHS 202781, V2LHS 201856
qPCR primer: human GAPDH	Thermo Fisher Scientific	4333764F
qPCR primer: human MAP4K4	Thermo Fisher Scientific	Hs00377415 m1
qPCR primer: human MINK1	Thermo Fisher Scientific	Hs01093259 m1
qPCR primer: human TNIK	Thermo Fisher Scientific	Hs00323234 m1
qPCR primer: mouse Col1a1	Thermo Fisher Scientific	Mm00801666 g1
qPCR primer: mouse Ctgf	Thermo Fisher Scientific	Mm01192932 g1
qPCR primer: mouse Fn1	Thermo Fisher Scientific	Mm01256744 m1
qPCR primer: mouse Postn	Thermo Fisher Scientific	Mm00450111 m1
qPCR primer: mouse Ubc	Thermo Fisher Scientific	Mm01201237 m1
siRNA: GFP, GGCTACGTCCAGGA GCGCACC	This paper	N/A
siRNA: rat MAP4K4, GGTTGAAAGT GATCTATGG	This paper	N/A

**Recombinant DNA**		

Plasmid: pAd-Easy-1	Bert Vogelstein	https://www.ncbi.nlm.nih.gov/pubmed/9482916
Plasmid: pCDNA3-FLAG-JNK1 APF	Roger Davis	https://www.ncbi.nlm.nih.gov/pubmed/8137421
Plasmid: pCIneo-HA-TNIK	Ken-ichi Kariya	https://www.ncbi.nlm.nih.gov/pubmed/15342639
Plasmid: pCL-puro-mU6	Zhou Songyang	https://www.ncbi.nlm.nih.gov/pubmed/15100233
Plasmid: pClneo-FLAG-TNIK	Ken-ichi Kariya	http://kenkyushadb.lab.u-ryukyu.ac.jp/html/100000748_en.html
Plasmid: pCR3.1-FLAG-MAP4K4	Tse-Hua Tan	https://www.ncbi.nlm.nih.gov/pubmed/9890973
Plasmid: pMD2.G	Didier Trono	Addgene 12259
Plasmid: pShuttleCMV	Bert Vogelstein	https://www.ncbi.nlm.nih.gov/pubmed/9482916
Plasmid: psPAX2	Didier Trono	Addgene 12260

**Software and Algorithms**		

Developer XD	Definiens AG	https://www.definiens.com/uploads/resources/collateral/Data-Sheet_Developer_Sep16.pdf
ImageJ	Fiji	https://imagej.net/Fiji
Prism	GraphPad Software Inc	https://www.graphpad.com/scientific-software/prism/
StatView	SAS Institute	https://sas-statview.software.informer.com/5.0/
ZEN 2012 (black edition)	Zeiss	https://www.zeiss.com/microscopy/int/downloads.html
FieldTemplater	Cresset BioMolecular Discovery Ltd	https://www.cresset-group.com/products/forge/fieldtemplater/
FieldScreen	Cresset BioMolecular Discovery Ltd	https://www.cresset-group.com/tag/fieldscreen/
NMR spectra processing	Jeol Delta NMR v5.0.4.4 MestRe-C 2.3a	https://www.jeol.co.jp/en/https://mestre-c-version-2-3a.updatestar.com/en

### Contact for Reagent and Resource Sharing

Requests should be directed to the Lead Contact, Michael D. Schneider, Imperial College London, at m.d.schneider@imperial.ac.uk.

### Experimental Model and Subject Details

#### Human subjects

Male and female human heart samples were generously provided under local ethical permission by Drs. Cris dos Remedios and Paul Allen (Sydney Human Heart Tissue Bank; University of Sydney ethics committee approval HREC #2012/2814), and by Dr. Guillermo Torre-Amione (The Methodist Hospital; Baylor College of Medicine ethics committee approval H-15240). Written informed consent was received from participants prior to inclusion in the study. Diseased hearts were obtained at the time of therapeutic transplantation. Healthy myocardial samples were obtained from prospective donors for whom no recipient was identified, overlapping the heart failure samples in age (donor: mean 43.2 y, N = 10, male 3, female 7; dilated cardiomyopathy: mean 44.3 y, N = 14 including 8 familial cases, male 10, female 4; hypertrophic cardiomyopathy: mean 43.3 y, N = 3, male 1, female 2; ischemic heart disease: mean 51.6 y, N = 7, male 6, female 1; adriamycin cardiomyopathy: mean 14.5 y, N = 2, male 2, female 0). No systematic difference was seen due to age or sex; however, the total number of subjects was small and not intended to permit demographic inferences. Tissue samples were flash frozen in liquid nitrogen immediately after extirpation of the heart.

#### Animals

All animal procedures were performed with UK Home Office approval (PL 70/6806, 70/7880) or US institutional review (Baylor College of Medicine, AN-3049). Animal work performed in the US conforms to the NIH Guide for the Care and Use of Laboratory Animals (DHS Publication No. 85-23, revised 1985). Animal work performed in the UK conforms to the UK Animals (Scientific Procedures) Act, 1986, incorporating Directive 2010/63/EU of the European Parliament. Procedures for the husbandry and housing of animals follow the recommendations of the Association for Assessment and Accreditation of Laboratory Animal Care and UK Code of Practice for the Housing and Care of Animals Bred, Supplied or Used for Scientific Purposes. The Imperial Hammersmith campus animal facilities comprise an SPF animal breeding facility (H2) and a clean facility for experimental surgery and physiology (H1). Biosecurity and pathogen exclusions are taken from Federation of European Laboratory Animal Science Associations (FELASA) health monitoring guidelines, and all animals are screened four times per year. Mice were housed in Allentown XJ individually ventilated cages, with Dates and bedding (ECO2) and a 12:12 light:dark cycle. Special Diet Services RM1 and RM3 chow were provided for maintenance and breeders, respectively, *ad libitum*. Environmental enrichments included small tunnels, chew blocks, and facial tissues. The maximum housing density for unoperated mice was 7 per cage if < 25 g and 5 per cage if ≥ 25 g. Following infarction, mice were typically housed singly, for monitoring. Similarly, animals at Baylor College of Medicine were housed in an SPF facility for breeding and maintenance and a clean satellite facility for surgery and physiology, with minor modifications: the light:dark cycle was 14:10, the diet was Purina Rodent Laboratory Chow 5001, and environmental enrichments were not used at the time. No animals were involved in previous procedures, drug administration, or testing. Healthy, immunocompetent, male or female mice, aged 8-10 weeks were investigated as follows. Except where noted, all transgenic experiments were performed using hemizygous mice in an isogenic FVB/N background.

Ischemia-reperfusion for exploratory biochemical studies was performed in 8-10 week-old FVB/N mice by ligation of the left anterior descending coronary artery (Michael et al., 1999). The ligature was tightened around the vessel and a superimposed 1 mm length of PE-10 polyethylene tubing. For reperfusion, the ligature and tubing were removed. Ischemia and reperfusion were substantiated by the induction of reversible myocardial blanching and electrocardiographic ST segment elevation. The control (“sham”) operation comprised anesthesia, thoracotomy, and placement of the ligature without occlusion.

Biomechanical stress was induced by partially occluding the transverse aorta in 8-10 week-old male mice (FVB/N: Figure S1; C57BL/6: Figure S3) (Sano et al., 2004). The control (“sham”) operation comprised anesthesia, thoracotomy, and placement of the ligature without occlusion. Only mice in which Doppler flow measurements confirmed moderate to severe occlusion (right-to-left carotid artery velocity ratio > 3.5) were analyzed subsequently. The heart weight/body weight ratio, used to verify effective constriction, increased 20% at 7 d and 35% at 14 d. Doppler echocardiography (Zhang et al., 2000), cardiac MRI (Stuckey et al., 2014), and pressure-volume loop analysis (Song et al., 2011) were performed as described.

For compound testing in myocardial infarction, 9-10 week-old CD-1 female mice were used instead, given strain-dependent differences in the pharmacokinetics of DMX-5804 (unpublished results), and rates for post-MI rupture in male mice as high as 49% (Gao et al., 2005). DMX-5804 and the vehicle control were each administered by oral gavage, as two doses (50 mg kg^-1^ in 1.5% Captisol), 10 hr apart. General anesthesia was induced with 4% isoflurane, then maintained at 2% in 100% O_2_. Mice were treated subcutaneously with 0.024 mg buprenorphine (average, 1.1 mg kg^−1^; Vetergesic, Alstoe Animal Health), intubated, and ventilated using a tidal volume of 250 μL and respiratory rate of 150 breaths min^−1^ (Hugo-Sachs MiniVent type 845; Harvard Apparatus). After a left thoracotomy in the fourth intercostal space, the pericardium was removed. A 6-0 polypropylene suture was used to ensnare the left anterior descending coronary artery (LAD) and tied against a short section of PE-50 polyethylene tubing for 45 min; blood flow was re-established by removing the tubing, releasing the ligature. Ischemia and reperfusion were substantiated by reversible distal blanching and ST segment elevation. Mice were allowed to recover in a heated chamber for 20 min, then moved to a normal holding cage with supplemental heat if necessary.

#### Transgenic mice

FLAG-tagged wild-type human MAP4K4 cDNA, provided by Dr Tse-Hua Tan (Yao et al., 1999), was cloned downstream of the 5.5-kb *Myh6* promoter (Subramaniam et al., 1991) for cardiomyocyte-restricted expression. The resulting construct was injected into the male pronucleus of FVB/N fertilized oocytes, and the injected zygotes were transferred to pseudopregnant females. Four independent *Myh6-Map4k4* founder lines were generated, with no premature lethality or other overt baseline phenotype. Results shown are from line 1998, with similar findings from other founders.

*Myh6-Gnaq* transgenic mice (Sakata et al., 1998) harbor the 1.46 kb wild-type mouse *Gnaq* cDNA cloned downstream of the *Myh6* promoter, and were engineered as above; the line Gαq-25, used here, was kindly provided by Dr Gerald Dorn. *Myh6-Tnfa* transgenic mice (MHCsTNF) (Sivasubramanian et al., 2001) express wild-type mouse TNFα driven by the *Myh6* promoter, engineered analogously, and were kindly provided by Dr Doug Mann.

#### Cell lines

Human iPSC-CMs were obtained from Cellular Dynamics (iCell; Figures 1 and 3) and Axiogenesis (CorV4.U; Figures 3 and 4) and cultured in the respective maintenance medium.

### Method Details

#### Cell culture

iCell cardiomyocytes were thawed, transferred to Plating Medium, counted using a Vi-CELL XR cell viability analyzer (Beckman Coulter), and seeded into 24-well plates for RNA collection (60,000 cells well^-1^; Greiner) or μClear half-area 96-well plates for gene silencing (10,000 cells well^-1^; Greiner). Plates were coated with 10 μg ml^-1^ collagen type I (BD Biosciences). Cells were cultured in iCell Cardiomyocytes Plating Medium for 2 d and in Maintenance Medium thereafter. For compound dose-response comparisons, the cells were deposited in Plating Medium at 10,000 cells well^-1^ in 0.1% (w/v) gelatin-coated 384-well white Greiner plates and were cultured as above for 8 d before treatment.

vCor.4U cardiomyocytes were cultured, except where noted, on white (viability) or black (FLIPR) clear-bottom 384-well plates, treated for 1 hr with 50 μM fibronectin (Sigma-Aldrich). To minimize batch to batch variation, thawed cells first were transferred from cryo-vials into sterile 250 mL flasks containing pre-warmed vCor.4U maintenance medium, for 3-5 d. The cells were then plated at 1000 well^-1^ for viability assays or 5000 cells well^-1^ for FLIPR experiments, all in 40 μl well^-1^ of the maintenance medium. Cells were cultured in Axiogenesis complete medium followed by incomplete medium, were subjected to oxidative stress ± test compounds on day 3, and were assayed on day 4 (Figures 4D and 4E). Alternatively, complete medium was replaced on day 2 of culture with 40 μl of DMEM high Glucose (no FCS or pyruvate), followed by removal and replacement of 20 μl every 2 d for 10 d (Figures 4A–4C).

H_2_O_2_, menadione (Sigma-Aldrich), doxorubicin (Calbiochem), and C2-ceramide (N-acetyl-D-sphingosine) were used at the indicated concentrations. MAP4K4 inhibitors were serially diluted in DMSO, transferred to an intermediate plate using assay medium as a diluting agent, and added at a final concentration of 0.1% DMSO. MAP4K4 inhibitors were added to cultured cardiomyocytes 1 hr before the cell death inducers, except where noted, and remained present for the duration of the experiment.

#### Western blotting

Cells and tissues were lysed in the presence of protease and phosphatase inhibitors (Pierce; Roche), resolved by SDS-polyacrylamide gel electrophoresis, and transferred to nitrocellulose membranes (Schleicher & Schuell; Optitran, GE Healthcare Life Sciences) for western blotting. Mouse monoclonal antibody to MAP4K4 was from Cell Signaling (ab56569). Rabbit antibodies to ERK, phospho-ERK (Thr202/Tyr204), JNK, phospho-JNK (Thr183/Tyr185), p38 and phospho-p38 (Thr180/Tyr182) were from Cell Signaling. Mouse antibody to the FLAG epitope (M2) was from Sigma-Aldrich. Mouse antibody to the HA epitope (F-7), rabbit antibody to caspase-3 (H-277) and HRP-conjugated goat antibody to total actin (I-19) were from Santa Cruz Biotechnology. Protein expression was visualized using horseradish peroxidase-conjugated secondary antibodies to IgG (Dako), followed by enhanced chemiluminescence reagents (Amersham; Pierce; Promega).

For initial studies (Figures S1 and S2), commercially available antibodies against MAP4K4 were inadequate and a polyclonal rabbit antibody was generated by a reported protocol (Yao et al., 1999). A synthetic peptide was synthesized, corresponding to amino acids 899–920 of mouse MAP4K4 (CNPTNTRPQSDTPEIRKYKKRFN), which are invariant in the rat and human kinases. For immunization, the peptide was coupled to keyhole limpet hemocyanin via the N-terminal cysteine. The resulting antibodies were affinity-purified on MAP4K4 peptide-conjugated Sepharose beads. Specificity was confirmed in HEK293T cells transiently transfected using Effectene (QIAGEN) and 1 μg of pCR3.1-FLAG-MAP4K4 (Yao et al., 1999), versus pCIneo-HA-TNIK or pClneo-FLAG-TNIK (TRAF2- and NCK-interacting kinase (Taira et al., 2004).

#### RNA interference

Human iPSC-CMs (iCell) were transduced with Hannon-Elledge microRNA-adapted shRNAs, a system optimized for efficient gene suppression (Silva et al., 2005). GIPZ shRNAmir sequences targeting the *MAP4K4* coding sequence (Open Biosystems) were incorporated into lentivirus using a second-generation packaging system from Didier Trono (psPAX2, pMD2.G). Thawed cells were cultured for 2 d in iCell Cardiomyocytes Plating Medium then for 5 d in *iCell* Cardiomyocytes Maintenance Medium. Cells were transduced for 6 hr (day 7), using serum-free DMEM, 8 μg ml^-1^ polybrene, a multiplicity of infection of 50, and non-silencing GIPZ (RHS4348) as the negative control. Cells were then cultured *for 2 d in iCell* Cardiomyocytes Maintenance Medium, and maintained for 2 d in low glucose DMEM containing 2% FBS. Cells were harvested for qPCR on day 11 or treated with H_2_O_2_ at the indicated concentrations, and assayed 24 hr later (day 12).

#### Quantitative real-time PCR (qRT-PCR)

RNA extraction was performed using RNAeasy Fibrous Tissue Mini Kits (QIAGEN) for mouse hearts and PureLink RNA Micro Scale Kits (Life Technologies) for cultured cells. RNA quality and quantity were assessed using a NanoDrop 1000 spectrometer (Thermo Fisher Scientific). RNA was converted to cDNA using High-Capacity cDNA Reverse Transcription Kits (Applied Biosystems). qRT-PCR was performed using TaqMan Gene Expression Assays, MicroAmp Optical 384-well plates, 2X TaqMan Gene Expression Master Mix, and a 7500 Real-Time PCR System (Applied Biosystems).

#### Immune complex kinase assays

MAP4K4 was precipitated using M2 antibody to the FLAG epitope, or antibody to endogenous MAP4K4, and protein G-Sepharose. Precipitates were washed twice in lysis buffer, twice with 500 mM LiCl, 100 mM Tris-HCl, pH 7.6, 0.1% Triton X-100, twice with kinase buffer (20 mM MOPS, pH 7.6, 2 mM EGTA, 10 mM MgCl_2_, 1 mM dithiothreitol, 0.1% Triton X-100, 1 mM Na_3_VO_4_), and were then mixed with 10 μg of myelin basic protein (Invitrogen) as substrate, 15 μM ATP, and 10 μCi [γ-^32^P]ATP in 30 mL of kinase buffer for 30 min at 30°C (Yao et al., 1999). His-MKK6 was used as the substrate for TAK1 (Zhang et al., 2000). Reaction mixtures were resolved by SDS-polyacrylamide gel electrophoresis, then were analyzed by western blotting and autoradiography.

#### Human cardiac muscle cell death

To measure loss of membrane integrity, iCell hiPSC-CMs were co-stained for 15 min at 37°C with 0.3 μM DRAQ7 (Biostatus), a cell-impermeable dye, and with 0.8 μM Hoescht 33342 (Molecular Probes), as the membrane-permeable dye. Dyes were removed and the nuclear fluorescence scored (typically, 15,000-20,000 cells well^-1^) using a Cellomics ArrayScan VTI High Content Screening platform. The line harbors a cardiomyocyte-specific *Myh6*-driven reporter gene for monomeric red fluorescent protein (RFP), enabling cell death to be scored exclusively in the myocyte population. Successfully transduced cells were identified on the basis of TurboGFP fluorescence. Images were analyzed with Developer XD, version 2.11 (Definiens).

#### Compound treatment

For cell-based dose-response experiments, MAP4K4 inhibitors were prepared in 96-well polypropylene plates as serial dilutions in DMSO of 10 mM stock solutions. To a sterile 96-well intermediate plate, 1 μl of each well was added to 99 μl of medium, the samples were mixed, and 5 μl well^-1^ from the intermediate plate were transferred to the cultured cells (final concentration, 0.1% DMSO; final top concentration of inhibitor, 10 μM). Assay plates were gently centrifuged then incubated for 1 hr at 37°C. The death triggers menadione, H_2_O_2_ and doxorubicin were prepared fresh on the day of treatment at 10x the final concentrations and 5 μl well^-1^ were added as appropriate. Assay plates were gently centrifuged and then incubated at 37°C for the duration of treatment.

#### CellTiter-Glo (CTG) luminescent cell viability assay

Assay plates were removed from the incubator, allowed to reach room temperature, inoculated with 20 μl well^-1^ CTG reagent (Promega), and gently agitated for 30 min. Prior to adding CTG, 2 μl well^-1^ of the culture medium was removed and placed into a white 384-well low volume plate for measurement of cardiac troponin release by AlphaLISA (below). Luminescence due to ATP, proportional to cell number, was captured on a PHERAstar Plus microplate reader (BMG Labtech). Normalized values were plotted against the log concentration of death signal or inhibitor, and a 4-parameter fit was used to identify IC_50_ and EC_50_, respectively.

#### Troponin I detection

Aliquots of medium collected after cells’ treatment with death signals and MAP4K4 inhibitors were incubated at room temperature with AlphaLISA human cardiac troponin I detection reagents (PerkinElmer): sequentially, anti-Troponin I acceptor beads for 1 hr, biotinylated antibody to troponin I for 1hr, and streptavidin (SA)-coated donor beads for 30 min. The AlphaLISA signal was acquired using the Pherastar Plus plate reader. All detection reagents were diluted in 1X AlphaLISA Immunoassay Buffer and a serially diluted analyte standard was used to quantitate the concentration of human cardiac troponin I (pg ml^-1^).

#### Hypoxia-reoxygenation assay

vCor.4U cells were plated at 10,000 well^-1^ in Cor.4U medium using black, clear-bottom 96-well plates pre-treated with Geltrex (GIBCO). After incubation overnight at 37°C, the media was replaced with phenol red-free DMEM (GIBCO) supplemented with 2% FCS, 2 mM L-glutamate, 1 mM pyruvate and 10 mM D-galactose. DMX-5804 prepared in DMSO and CellTox Green (Promega) were added and incubated for 1 hr at 37°C. A breathable seal (AeraSeal) was added to the plate before transferring to a CLARIOstar plate reader (BMG Labtech) equipped with an atmospheric control unit. Oxygen concentration ramping was executed as follows (% O2, hr), at 37°C and 5% CO_2_. Fluorescence measurements (λ_ex/em_ 483-14/530-30) were taken using the multiple scan and direct bottom features (5 positions read), and matrix averaging of each position every 20 min. Data were blank corrected, averaged (n = 2) and subjected to exponential smoothing with a damping factor of 0.3.

#### Ion channel safety screening

Electrophysiological recordings (Apconix) were performed using a Chinese Hamster Ovary cell line stably expressing full length hERG or hNaV1.5, or HEK293 cells stably expressing full length hCaV1.2. Single cell currents were measured in whole-cell configuration at 21-23°C using a Patchliner (Nanion Technologies). The internal solution for hERG contained (mM): 120 KF, 20 KCl, 10EGTA, 10 HEPES (pH 7.3). The internal solution for hNaV1.5 and CaV1.2 contained (mM): 140 CsF, 1 EGTA, 10 NaCl, 10 HEPES (pH 7.3). The external solution contained (mM): 138 NaCl, 4.5 KCl, 1.8 CaCl_2_, 1.0 MgCl_2_, 10 HEPES, 10 glucose (pH7.4). Late Na^+^ currents were activated with 50 μM veratridine. Cells were clamped at a holding potential of −80 mV before a depolarising step appropriate for each channel. Currents measured from the step were referenced to the holding current. Compounds were then incubated for 2-3 min prior to a second measurement using an identical pulse train.

#### Ames genotoxicity reverse mutation assay

Approximately 10^7^ bacteria were exposed in triplicate to the test agent (six concentrations, 7.8-250 μg ml^-1^), a vehicle control (vehicle) and a positive control for 90 min in low-histidine medium (sufficient for about 2 doublings.) The strains used were S. typhimurium TA98 (hisD3052, rfa, uvrB / pKM101; detects frameshift mutations) and TA100: hisG45, rfa, uvrB / pKM101; detects base-pair substitutions). The cultures were then diluted into indicator medium lacking histidine, dispensed into 384-well plates, and incubated for 48 hr at 37°C. The test was performed in the absence and presence of S9 metabolic activation, to identify pro-mutagens as well as directly acting mutagens. Only cells that underwent reversion grow, resulting in a color change. A two-fold increase over the vehicle control in the number of colonies indicates a positive response.

#### *In vitro* MAP4K4 kinase activity assay

MAP4K4 kinase activity was monitored using the CisBio HTRF Transcreener ADP assay, a competitive immunoassay with a reproducible Z’ > 0.6. In the detection step, endogenous ADP and d2-labeled ADP compete for binding an anti-ADP monoclonal antibody labeled with Eu^3+^ cryptate. A ratiometric fluorescent readout is used at 665 and 620 nm. Reactions were performed in the presence of 1% DMSO with ATP added at K_m_ (10 μM), 0.5 nM human MAP4K4 kinase domain (Invitrogen), 1 μM biotin-myelin basic protein as substrate (Invitrogen), and extension of reaction time to 2 h. Assays were run in Greiner low volume plates with a final reaction volume of 10 μl. Percent inhibition was calculated from 100% activity of MAP4K4 (DMSO only) and 0% activity (no enzyme added). Data were either reported as % inhibition at a single dose or as pIC_50_ values derived from dose response curves (pIC_50_ = -log[IC_50_]).

#### Computational chemistry

**(a) Hardware and software.** The Field Point Pharmacophore was created using the Mac OS X 10.6 version of FieldTemplater v. 2.1.1 running on a Mac Pro (3x2.97 GHz 4-Core Intel Xeon). The virtual screen was run at Cresset BioMolecular Discovery Ltd (https://www.cresset-group.com). Blaze v.10.0 was mounted on a Linux (Debian) Cluster containing 50 Intel and AMD nodes and was used with the input seed field to search 370M field patterns from 3.7M commercially available molecules, 100 conformations each. Each search took 12-15 CPU hours, returning a field similarity with the seed input for every molecular entry. The 200 with highest similarity were used as the virtual screening “hit list” in subsequent analysis and acquisition. Overlays were examined using Forge v.10.0 on Macintosh or PC desktops.

**(b) Creation of the field point pharmacophore.** FieldTemplater v. 2.11 was used to derive the field point pharmacophore for the template compounds, using the inbuilt conformation hunter and XED molecular mechanics force fields. Using an upper threshold of 6 kcal mol^-1^ above the calculated global energy minimum gave 2 conformations of PD-407824; 4 of GW5074; and 1 of Kenpaullone, reflecting the low number of rotatable bonds in these molecules. Each conformation was then equipped with four types of molecular field points: the extrema of positive and negative electrostatic regions, hydrophobic regions, and areas of maximum van der Waals attraction. The field points were calculated in terms of the interaction of appropriate charged and neutral probes at and beyond the molecular surface and were visualized as colored spheres at the position of the extrema, sized according to their magnitude. Each conformation was compared in turn to all those of another molecule. Initial alignments were generated on the basis of distances, sizes and types of field point and were grouped into “cliques,” whose scores reflect the size of clique and the field points within it. For top scoring alignments, the field points of one conformation were used to sample the full field of the second molecule, and the process was then repeated for the second on the first. The average of the two scores was taken as the score for that pair, normalized to give a Dice field similarity metric, and the process was repeated over the complete set of compounds. For the three template compounds here, FieldTemplater identified five potential solutions, one of which was used as the seed for virtual screening.

**(c) Virtual Screening with FieldScreen.** Four screens were run, one with the complete template and a further three independent searches with the individual components, credibly the bioactive conformations of each. In each case, the relevant field point pattern was compared to those of 100 conformations each from 3.7M commercially available molecules. The scoring function reflects the electrostatic and steric similarity of compounds within the database to those of the seed pharmacophore and was used to rank the virtual hits. The 40 top available compounds were purchased from the Enamine Screening Collection (https://enamine.net) or Chembridge Express Pick (https://www.chembridge.com), were validated by ^1^H nuclear magnetic resonance and mass spectroscopy, and were assessed for activity against human MAP4K4 by the HTRF Transcreener ADP assay.

#### Selectivity and microsomal stability assays

Kinase selectivity profiling was performed by the International Centre for Kinase Profiling, MRC Protein Phosphorylation and Ubiquitylation Unit (141 kinases; Premier Screen, http://www.kinase-screen.mrc.ac.uk), using a [γ-^33^P]ATP filter binding assay (Bain et al., 2007). Compounds were screened at 1 μM. For key hits, pIC_50_ values were then determined by HTRF as above. More complete kinome profiling was performed by Reaction Biology (376 kinases). Stability in mouse and human liver microsomes was determined by Cyprotex.

#### Analytical chemistry

Compounds were characterized by liquid chromatography–mass spectrometry (LC-MS) using Method A or B below and/or nuclear magnetic resonance (NMR).

Method A: Phenomenex Luna C18(2), 3 μm, 50 × 4.6 mm; A = water + 0.1% formic acid; B = MeOH + 0.1% formic acid; 45°C; %B: 0.0 min 5%, 1.0 min 37.5%, 3.0 min 95%, 3.5 min 95%, 3.51 min 5%, 4.0 min 5%; 2.25 mL min^-1^.

Method B: Phenomenex Gemini NX-C18, 5 μm, 150 × 4.6 mm; A = water + 0.1% formic acid; B = MeOH + 0.1% formic acid; 40°C; %B: 0.0 min 5%, 0.5 min 5%, 7.5 min 95%, 10.0 min 95%, 10.10 min 5%, 13 min 5%; 1.5 mL min^-1^.

NMR spectra were obtained on Bruker Advance 400, Bruker DRX 400 or Jeol 400 ECS NMR spectrometers at room temperature unless otherwise stated. ^1^H NMR spectra are reported in ppm and referenced to the residual solvent peaks e.g., DMSO-d_6_ (2.50 ppm), CDCl_3_ (7.26 ppm) or CD_3_OD (3.31 ppm).

#### Preparative High Performance Liquid Chromatography (HPLC)

Mass-directed purification was performed to isolate DMX-5804 and a number of the intermediates, using preparative reversed-phase HPLC-MS (Phenomenex Luna C18(2), 5 μm, 100 × 21.2 mm) and pH ∼2 as below.

Method C: A = water + 0.025% formic acid; B = acetonitrile, 30°C; %B: 0.0 min Initial 10%, 10.0 min 20%, 20.0 min 30%, 30.0 min 50%, 40.0 min 75%; 40.0 mL min^-1^.

Method D: A = water + 0.1% formic acid; B = MeOH + 0.1% formic acid; 20°C; %B: 0.0 min Initial 45%, 0.1 min % as per Initial, 7.0 min 85%, 9.0 min 95%, 10.0 min 95%, 10.1 min back to Initial %; 12.0 min Initial %; 20.0 mL min^-1^.

Synthesis of 5-[4-(2-Methoxy-ethoxy)-phenyl]-7-phenyl-3,7-dihydro-pyrrolo[2,3-d]pyrimidin-4-one (DMX0005804)

See Methods S1 for illustrations of synthetic route and key structures.

#### Step 1: 4-Chloro-5-iodo-7-phenyl-7H-pyrrolo[2,3-d]pyrimidine (3)

To a solution of 4-chloro-5-iodo-7H-pyrrolo[2,3-d]pyrimidine (1) (15.0 g, 53.5 mmol) in dimethylformamide (100 mL) was added 2-phenyl-1,3,2-dioxoborinone (2) (17.3 g, 107.0 mmol), copper (II) acetate monohydrate (21.4 g, 107.0 mmol), and activated molecular sieves (4Å, 0.4 g), followed by addition of triethylamine (22.3 mL, 160.4 mmol). The resulting reaction mixture was stirred at 60°C for 24 hr then cooled to room temperature and the solvent concentrated *in vacuo*. The crude residue was dissolved in dichloromethane (300 mL) and quenched with saturated ethylenediaminetetraacetic acid (EDTA) solution (100 mL). The separated aqueous layer was extracted with dichloromethane (2 × 100 mL), then the combined organic layer was dried over anhydrous sodium sulfate, filtered, and concentrated *in vacuo*. The crude compound was purified by reversed-phase preparative HPLC-MS (Method C) to afford 4-chloro-5-iodo-7-phenyl-7H-pyrrolo[2,3-d]pyrimidine (3) as an off-white solid (6.2 g, 33%).

^1^H NMR (400 MHz, DMSO-*d*_*6*_): δ 8.70 (s, 1H), 8.39 (s, 1H), 7.81-7.77 (m, 2H), 7.61-7.56 (m, 2H), 7.47 (tt, *J =* 7.8, 1.4 Hz, 1H); LC-MS. R_t_ 3.37 min (Method A); (ESI^+^) m/z 356, 358 [M+H]^+^.

#### Step 2: 5-Iodo-7-phenyl-3,7-dihydro-pyrrolo[2,3-d]pyrimidin-4-one (4)

A suspension of 4-chloro-5-iodo-7-phenyl-7H-pyrrolo[2,3-d]pyrimidine (4.0 g, 11.3 mmol) and sodium acetate (1.9 g, 22.5 mmol) in acetic acid (25 mL) was heated at 100°C for 15 h. The reaction mixture was concentrated *in vacuo*. The crude solid was diluted with water and the resulting solid was filtered and dried under vacuum to afford 5-Iodo-7-phenyl-3,7-dihydro-pyrrolo[2,3-d]pyrimidin-4-one (4) as a yellow solid (3.68 g, 97%).

^1^H NMR (400 MHz, DMSO-*d*_*6*_): δ 12.16 (br s, 1H), 7.95 (s, 1H), 7.70-7.66 (m, 2H), 7.68 (s, 1H), 7.56-7.51 (m, 2H), 7.41 (tt, J = 7.3 1.4 Hz, 1H); HPLC-MS. Rt 2.79 min, (Method A); (ESI+) m/z 338 [M+H]^+^.

#### Step 3: 5-[4-(2-Methoxy-ethoxy)-phenyl]-7-phenyl-3,7-dihydro-pyrrolo[2,3-d]pyrimidin-4-one (DMX0005804)

A mixture of 5-iodo-7-phenyl-3,7-dihydro-pyrrolo[2,3-d]pyrimidin-4-one (4) (180 mg, 0.534 mmol), 2-(4-(2-methoxyethoxy)phenyl-4,4,5,5-tetramethyl-1,3,2-dioxaborolane (5) (186 mg, 0.67 mmol), Pd(dppf)Cl_2_ (43.6 mg, 0.05 mmol) and potassium carbonate (148 mg, 1.07 mmol) in dioxane:H_2_O (3 mL, 9:1) was de-oxygenated for 5 mins then heated in a microwave reactor at 120°C for a total of 90 min. The reaction was repeated on the same scale with heating in a microwave for 2 h. The combined reaction mixture was filtered through celite 545 and washed with methanol. The filtrate was concentrated *in vacuo*. The crude solid was diluted with dichloromethane and water and the layers separated via a phase separator cartridge. The combined organics were concentrated *in vacuo*. The crude solid was purified by silica gel chromatography, eluting with 0%–7.5% methanol/dichloromethane, followed by reversed-phase preparative HPLC-MS (Method D). A final purification using silica gel chromatography was carried out by eluting with 0%–5% methanol/dichloromethane to afford 5-[4-(2-Methoxy-ethoxy)-phenyl]-7-phenyl-3,7-dihydro-pyrrolo[2,3-d]pyrimidin-4-one (DMX0005804) as a white solid (70 mg, 18%).

^1^H NMR (400 MHz, DMSO-*d*_*6*_): 12.13 (br s, 1H), 7.95 (s, 1H), 7.93 (d, *J =* 8.8 Hz, 2H), 7.78-7.75 (m, 2H), 7.73 (s, 1H), 7.58-7.55 (m, 2H), 7.42 (tt, *J =* 7.6, 1.3 Hz, 1H), 6.95 (d, *J =* 8.8 Hz, 2H), 4.13-4.11 (m, 2H), 3.69-3.66 (m, 2H), 3.32 (s, 3H); ^13^C NMR (400 MHz, DMSO-*d*_*6*_): 158.6, 157.2, 147.7, 144.3, 137.2, 129.5, 129.1, 127.0, 125.8, 124.5, 120.7, 120.6, 113.9, 106.0, 70.4, 66.8, 58.1; LC-MS. R_t_ 7.66 min (Method C); (ESI^+^) m/z 362 [M+H]^+^.

#### Human cardiac muscle cell function

Calcium transients in hiPSC-CMs were assessed using FLIPR Tetra instrumentation and Early Tox Cardiotoxicity kits (Molecular Devices). vCor.4U cells were subjected to graded concentrations of menadione and the MAP4K4 inhibitors as described for the viability assays. Compound concentrations and time course are denoted in the figure and figure legend. Vehicle controls were used throughout. Cells were incubated for 2-3 hr at 37°C in 5% CO_2,_ using 3 μL well^-1^ of EarlyTox Cardiotoxicity Kit concentrate as the fluorescent calcium indicator dye. Plates were then removed from the incubator and allowed to reach room temperature. Intracellular calcium oscillations were monitored using a FLIPR Tetra system with excitation LED bank 470-495 nm and emission filter set 515-575 nm (Molecular Devices). Fluorescence intensity signals were acquired for 600 reads at 1.2 ms intervals. Beat frequency, median peak height, median peak width and total peak area were calculated and compared with the baseline control and with menadione alone. Protection of calcium cycling by DMX-4804 was confirmed in 3 independent experiments with overlapping conditions, and results are shown for a full dose-response study, analyzing 4 wells for each point.

Mitochondrial function in hiPSC-CMs was determined using a Seahorse XFe24 Analyzer. vCor.4U cells (60,000 well^-1^) were transferred to 0.1% gelatin-coated XF24 plates and maintained for 5 d, as described above. On day 6, DMX-5804 was added 45 min prior to challenge with menadione for 2 h. The medium was replaced 1 hr before the assay, using bicarbonate-free Seahorse assay medium (8.3 g L^-1^ DMEM Base (Sigma-Aldrich), 10 mM glucose, 2 mM L-alanyl-L-glutamine dipeptide (Glutamax-1, GIBCO), 1 mM sodium pyruvate (GIBCO), pH 7.4). Cells were maintained at 37°C without supplemental CO_2_ starting 1 hr before the assay (IL10 incubator, VWR). For each state measured, three assay cycles were performed (4 min mixing, 2 min wait, and 2 min measurement periods, with readings every 15 s). The basal oxygen consumption rate (OCR) and extracellular acidification rate (ECAR) were determined, followed by sequential injection of 1 μM oligomycin A (Abcam) to inhibit ATP synthase, 0.5 μM carbonyl cyanide-4-phenylhydrazone (FCCP; Abcam) to uncouple oxidative phosphorylation, and 1 μM antimycin A/rotenone (Sigma, Abcam) to inhibit mitochondrial complex III and I. For each condition, 12 wells were tested, comprising 4 independent experiments.

#### Human engineered heart tissue

Fibrin-based hEHTs were generated between flexible silicone posts as described (Breckwoldt et al., 2017, Hansen et al., 2010), with minor modifications. 1.8 mL well^-1^ of 2% agarose in PBS was pipetted into 24-well plates, Teflon spacers (EHT Technologies) were placed inside each well as a casting mold, and the agarose was left to set for 15 min. The spacers were removed from each well, and a pair of polydimethylsiloxane posts was inserted from above (EHT Technologies: Young’s modulus 1.7 mPa, length 10 mm, radius, 0.5 mm). VCor.4U cells (4 × 10^6^) were resuspended in 400 μL of master mix (340 μL DMEM (Biochrom), 10% heat-inactivated fetal calf serum (GIBCO), 2 mM glutamine (Life Technnologies), 1% penicillin/streptomycin (GIBCO); 60 μL 2x DMEM (GIBCO), 20% horse serum (GIBCO), 2% penicillin/streptomycin). Bovine fibrinogen (Sigma) was added (10 μL: 200 mg mL^-1^, 95 μg mL^-1^ aprotinin (Sigma), 0.9% NaCl), and 100 μL aliquots were transferred to PCR tubes containing 3 μL of 100 U ml^-1^ thrombin (Biopur) in 60% PBS. Cell suspensions then were immediately transferred to the agarose casting molds. After 90 min to allow solidification, 300 μL well^-1^ of EHT medium was added (DMEM, 10% horse serum, 0.1% human insulin (Sigma), 0.1% aprotinin), to hydrate the gels for a further 50 min. The hEHTs were then transferred to fresh 24-well plates with 1.5 mL well^-1^ of EHT medium, which was replaced every Monday, Wednesday, and Friday for 12 d. Auxotonic force measurements were performed daily by optical tracking (Hansen et al., 2010) and calculated on the basis of post deflection distance (δ), post length (L), post radius (R), and the elastic modulus (E), using the formula F = 3πER^4^δ4L^-3^ (Schaaf et al., 2011) and the indicated post parameters. Beating rate and force generation increased between d 5 and 10 and were stable for at least 7 d thereafter. On day 12, the hEHTs were challenged for 24 hr with menadione, in the absence or presence of DMX-5804. Medium, including DMX-5804, was replenished daily. For each time-course, 4 constructs were tested, comprising 4 independent experiments.

#### Oral PK

*In vivo* pharmacokinetic profiling was performed in female CD-1 mice, using 3 animals per time point. First, 30% w/v Kleptose as excipient was dissolved in water and vortexed gently for several min. Next, 30 mg of the test compound was dissolved into 0.6 mL of DMSO, for a concentration of 50 mg ml^-1^, and 4.5 mL of the Kleptose solution was added to 0.5 mL of the test compound solution. A precipitate forms, which re-dissolves over 2-5 min, leaving a clear or slightly hazy solution with a final concentration of 5 mg ml^-1^. The dosing solution is used as soon as practicable, vortexing immediately prior to use. This amount was sufficient formulation for 25 doses of 200 μL (20 g mouse). Compounds were administered orally at 50 mg kg^-1^, with terminal blood (plasma) sampling at 10 min, 30 min, 1 h, 2.5 h, 5 h, 10 h, and 20 h.

#### Target engagement assays

Lysates were prepared and assayed using a desthiobiotin-ATP probe Kinase Enrichment Kit (Thermo Fisher Scientific) (Patricelli et al., 2011). In brief, tissue samples (100-120 mg) were cut into small pieces and resuspended in 1 mL of Lysis Buffer containing protease and phosphatase inhibitors. After homogenization (30 s on ice), the lysates were centrifuged (16,000 x g at 4°C for 10 min) and a buffer-exchange to Reaction Buffer was performed, to remove endogenous ATPs. Protein concentrations were measured by the BCA assay (Thermo Fisher Scientific). Compounds were added to 500 μL of lysate at 2 mg/ml in presence of 20 mM MgCl_2_ and incubated for 10 min at room temperature. The ActivX desthiobiotin-ATP probe (5 μM) was added, and samples were incubated for 10 min. Reactions were stopped by adding 500 μL of 8 M Urea/Lysis Buffer, and labeled kinases were captured by incubating samples with 60 μL of 50% Streptavidin-Agarose beads. Samples were incubated with constant inversion for 2 hr at room temperature. Samples were centrifuged (1000 x g for 1 min) and washed three times with 500 μL of 4 M Urea/Lysis Buffer. Captured kinases were eluted by boiling in 30 μL of Laemmli sample buffer for 15 min. Samples were analyzed by western blotting for MAP4K4 and for Src or p38 and were normalized to the capture of the untargeted control kinase.

#### Histology

To determine infarct size and area at risk, mice were re-intubated after 24 hr of reperfusion, ventilated as previously, and a lateral thoracotomy was performed to expose the myocardium. The suture around the LAD was re-occluded and 1 mL of 1% Evan blue solution in saline was infused via the right ventricle. Hearts were rapidly excised, rinsed in PBS and allowed to freeze at −20°C for 20 min. Short axis sections were prepared (1 mm), incubated in 2% 2,3,5-triphenyltetrazolium chloride (TTC) for 15 min, placed in 4% paraformaldehyde overnight, and rinsed in PBS for 15 min before imaging. AAR was calculated as 100% minus the Evans blue-positive area.

For other histological studies, hearts were pressure-perfused with formalin, dehydrated to 70% ethanol, mounted in paraffin, sectioned, and stained with hematoxylin and eosin or picrosirius red. For immunostaining, sections were de-paraffinized, dehydrated, washed with PBS, and treated with 0.4% Triton-X in PBS. Nuclei were stained with 2.5 μg ml^-1^ diamidinophenolindole (DAPI). Alternatively, fixed frozen sections were used. Cleaved DNA (apoptotic cells) was visualized using TUNEL ApopTag Kits (Millipore). Cardiomyocytes were identified using MF20 antibody to sarcomeric myosin heavy chains (MyHC; R&D Systems) (fluorescein-conjugated: Figure S3C; unconjugated, plus Alexa Fluor-555 anti-mouse IgG: Figure S3H; conjugated and unconjugated mouse IgG_2b_ were the respective controls). Sections were counterstained with DAPI and treated with Prolong Gold Antifade Mountant (Life Technologies). Images were captured with a Zeiss Axioplan 2 epifluorescence microscope, LSM-510 inverted confocal microscope, or LSM-780 inverted confocal microscope, using Zeiss ZEN 2012 (black edition) and ImageJ (Fiji) software.

#### Rat cardiac muscle cell death

Ventricular myocytes from 1 to 3 day-old Sprague-Dawley rats were enzymatically dissociated, purified by Percoll gradient centrifugation and pre-plating, and cultured as described (Oh et al., 2003). To detect hypodiploid DNA, flow cytometry was performed (XL or LSRII, BD Biosciences), using propidium iodide to measure DNA content and sampling > 5000 cells for each histogram. Myocyte identity was confirmed using FITC-conjugated MF-20 antibody to sarcomeric MyHC (R&D Systems). To detect dissipation of mitochondrial membrane potential (ΔΨm), cells were incubated for 60 min in 5 μg ml^-1^ 5, 5′, 6, 6’-tetrachloro-1, 1’, 3, 3′-tetraethylbenzimidazolyl carbocyanine iodide (DePsipher; R&D Systems). When ΔΨm is intact, mitochondrial uptake and aggregation of the dye result in red fluorescence; when ΔΨm is disturbed, the dye diffuses to the cytoplasm and reverts to its monomeric green form. Myocyte identity was confirmed using mouse antibody to sarcomeric tropomyosin (Sigma-Aldrich) conjugated with Texas Red-X (Molecular Probes).

#### Adenoviruses

Using pAd-Easy-1 and pShuttleCMV (B. Vogelstein, Johns Hopkins University), we created recombinant adenoviruses expressing wild-type MAP4K4, dominant-negative mutations of MAP4K4 (K54E, K54R), dominant-negative TAK1 (K63W), and dominant-negative JNK1 (APF). MAP4K4 is alternatively spliced, with the presence or absence of an SH3-like domain being one potentially important difference. Here, we used the shorter form, which predominates in cardiac muscle (not shown) and other cell types (Yao et al., 1999). Kinase-inactive mutations of MAP4K4 were generated by site-directed mutagenesis using wild-type human MAP4K4 cDNA, with the FLAG epitope, as template (Yao et al., 1999). Dominant-negative, FLAG-tagged JNK1 (JNK1 APF) cDNA was provided by R. Davis (University of Massachusetts). Adenovirus encoding dominant-negative p38α (TGY→AGF) with a FLAG epitope tag was provided by Y. Wang (University of California, Los Angeles), and adenovirus encoding Bcl2 by L. Kirshenbaum (University of Manitoba). Rat ventricular cardiomyocytes were infected at a multiplicity of infection of 10 for each virus, then cultured in serum-free medium for 24-48 h. Multiplicity of infection was kept constant in each experiment using control virus containing CMV-GFP.

For RNA interference in rat cardiomyocytes, an irrelevant siRNA sequence against GFP (5′-GGCTACGTCCAGGAGCGCACC-3′) was obtained and siRNA against rat MAP4K4 was designed using ON-TARGET (both, Dharmacon). Of four sequences tested, one 5′-GGTTGAAAGTGATCTATGG-3′ achieved efficient knockdown of co-transfected MAP4K4 (Figure S4B, left). The sequence was synthesized as a hairpin structure 5′-GATCTTTggttgaaagtgatctatggTTCAAGAGAccatagatcactttcaaccTTTTTGGA-3′ and inserted in a modified pShuttle vector with a MU6 promoter (Z. Songyang, Baylor College of Medicine) (O’Connor et al., 2004). Adenoviruses expressing the siRNAs were generated using AdEasy-1. Cardiomyocytes were infected as above for 36 h.

### Quantification and Statistical Analysis

Data are reported as the mean ± standard error, using a significance level of p < 0.05. The number of replicates is indicated in the figure legends; “N” denotes the number of independent experiments and ”n” denotes the number of cultures or number of animals, as appropriate. Data were analyzed by two-way ANOVA, using Scheffe’s or Bonferroni test for multiple comparisons and Welch’s t test for pairwise comparisons (StatView 5.0, Abacus Concepts; Prism 5-7, GraphPad). MRI and pressure-volume loops were analyzed by one-way ANOVA and Neuman-Keuls test. Survival probabilities were analyzed by the Kaplan-Meier method.

### Data and Software Availability

The accession number for the chemical structure of DMX-5804 reported in this paper is PubChem 98666.

## Supplemental Information

Document S1. Figures S1–S5, Table S1, and Methods S1

Table S2. Mean Percentage Residual Activity Is Shown in the Presence of the Indicated Inhibitors at 1 μM (Dundee) or 0.09 μM (30× the IC_50_ against MAP4K4; Reaction Biology), Related to Figure 2 Values <50% are highlighted (shaded). N.d., not determined.

Document S2. Article plus Supplemental Information
