# MAP4K4 Inhibition Promotes Survival of Human Stem Cell-Derived Cardiomyocytes and Reduces Infarct Size *In Vivo*

**DOI:** 10.1016/j.stem.2020.01.015

**Published:** 2020-03-05

**Authors:** Lorna R. Fiedler, Kathryn Chapman, Min Xie, Evie Maifoshie, Micaela Jenkins, Pelin Arabacilar Golforoush, Mohamed Bellahcene, Michela Noseda, Dörte Faust, Ashley Jarvis, Gary Newton, Marta Abreu Paiva, Mutsuo Harada, Daniel J. Stuckey, Weihua Song, Josef Habib, Priyanka Narasimhan, Rehan Aqil, Devika Sanmugalingam, Robert Yan, Lorenzo Pavanello, Motoaki Sano, Sam C. Wang, Robert D. Sampson, Sunthar Kanayaganam, George E. Taffet, Lloyd H. Michael, Mark L. Entman, Tse-Hua Tan, Sian E. Harding, Caroline M.R. Low, Catherine Tralau-Stewart, Trevor Perrior, Michael D. Schneider

(Cell Stem Cell *24*, 579–591.e1–e12; April 4, 2019)

In the originally published version of our manuscript, one of the authors was incorrectly listed as “Narasimham, P.” To address this, we have now corrected the spelling of the name both here and in our original article online to “Narasimhan, P.” We apologize for the oversight and for any resulting confusion.

