# Supplementary material for: MAP4K4 Inhibition Promotes Survival of Human Stem Cell-Derived Cardiomyocytes and Reduces Infarct Size In Vivo
Source: Cell Stem Cell. 2019 Apr 4;24(4):579–591.e12. doi: 10.1016/j.stem.2019.01.013 (PMC6458995; doi:10.1016/j.stem.2019.01.013)
Supplement: Document S1. Figures S1–S5, Table S1, and Methods S1 [file mmc1.pdf]

Cell Stem Cell, Volume *24*

Supplemental Information

MAP4K4 Inhibition Promotes Survival of Human Stem Cell-Derived Cardiomyocytes and Reduces Infarct Size *In Vivo*

Lorna R. Fiedler, Kathryn Chapman, Min Xie, Evie Maifoshie, Micaela Jenkins, Pelin Arabacilar Golforoush, Mohamed Bellahcene, Michela Noseda, Dörte Faust, Ashley Jarvis, Gary Newton, Marta Abreu Paiva, Mutsuo Harada, Daniel J. Stuckey, Weihua Song, Josef Habib, Priyanka Narasimham, Rehan Aqil, Devika Sanmugalingam, Robert Yan, Lorenzo Pavanello, Motoaki Sano, Sam C. Wang, Robert D. Sampson, Sunthar Kanayaganam, George E. Taffet, Lloyd H. Michael, Mark L. Entman, Tse-Hua Tan, Sian E. Harding, Caroline M.R. Low, Catherine Tralau-Stewart, Trevor Perrior, and Michael D. Schneider

MAP4K4 inhibition promotes survival of human stem cell-derived cardiomyocytes and reduces infarct size in vivo

Lorna R. Fiedler, Kathryn Chapman, Min Xie, Evie Maifoshie, Micaela Jenkins, Pelin Arabacilar Golforoush, Mohamed Bellahcene, Michela Noseda, Dörte Faust, Ashley Jarvis, Gary Newton, Marta Abreu Paiva, Mutsuo Harada, Daniel J. Stuckey, Weihua Song, Josef Habib, Priyanka Narasimham, Rehan Aqil, Devika Sanmugalingam, Robert Yan, Lorenzo Pavanello, Motoaki Sano, Sam C. Wang, Robert D. Sampson, Sunthar Kanayaganam, George E. Taffet, Lloyd H. Michael, Mark L. Entman, Tse-Hua Tan, Sian E. Harding, Caroline M. R. Low, Catherine Tralau-Stewart, Trevor Perrior, & Michael D. Schneider

SUPPLEMENTARY INFORMATION

Figure S1, related to Figure 1

Figure S2, related to Figure 1

Figure S3, related to Figure 1

Figure S4, related to Figure 1

Figure S5, related to Figures 3 and 4

Table S1, related to Figure 1

Table S2, related to Figure 2 (provided separately as Excel table).

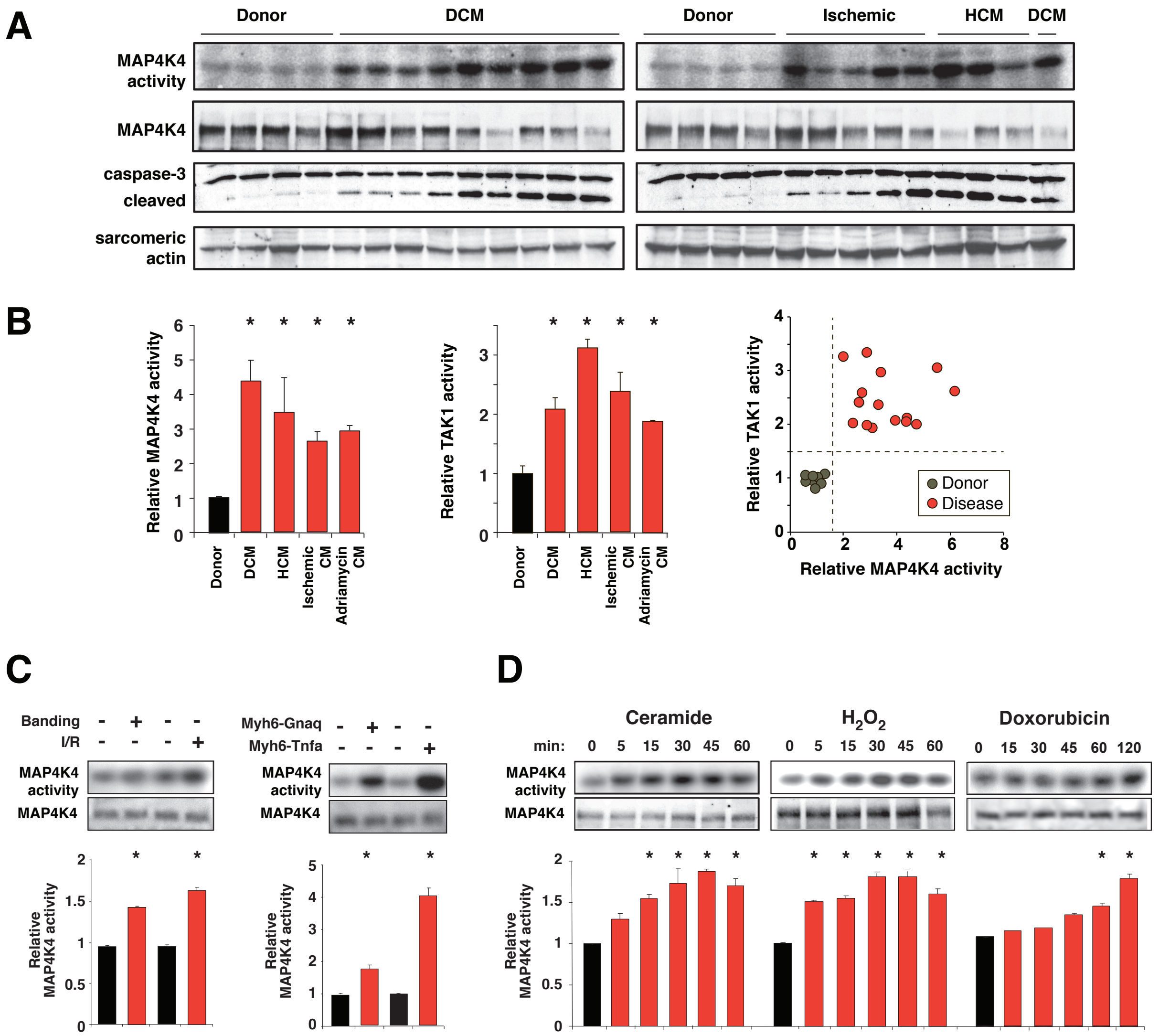

Fig. S1, related to Figure 1. MAP4K4 activation by diverse cardiac death signals and its prevalence in human heart failure. (**A, B**) Activation of endogenous human MAP4K4 in diseased human myocardium. (**A**) MAP4K4 activity was measured by the immune complex kinase assay. Total MAP4K4, caspase-3 cleavage, and α-sarcomeric actin as control for sample loading and myocyte content were visualized by Western blotting. Donor, healthy hearts; DCM, dilated cardiomyopathy; HCM, hypertrophic cardiomyopathy. Activation of human cardiac MAP4K4 was prevalent in chronic heart failure from diverse etiologies (N = 26), relative to healthy donor hearts (N = 10), associated with active (cleaved) caspase-3. (**B**) Activation of MAP4K4 (left) and TAK1/MAP3K7 (center), by immune complex kinase assays. See Methods for the patient characteristics. N ≥ 5 (excepting adriamycin cardiomyopathy, N = 2); *, P < 0.002. Right, MAP4K4 and TAK1 activities show no overlap between healthy hearts and failing (double-positive) ones, using a threshold of 1.5x the healthy mean. (**C**) Activation of endogenous MAP4K4 in diseased mouse myocardium. Mice were subjected to pathophysiological (left) or genetic (right) interventions, and MAP4K4 activity was analyzed as in panel A. I/R, ischemia-reperfusion, 30 min/2 hr; aortic banding, 7 d; age, 8 or 10 weeks, respectively. n = 3; *, P < 0.003. (**D**) Activation of endogenous MAP4K4 in cultured rat cardiomyocytes. MAP4K4 activity was measured as in panel A, after treatment with C2-ceramide, H_2O_2, or doxorubicin for the intervals shown. n = 3; *, P ≤ 0.03. All numerical results are shown as the mean ± SE.

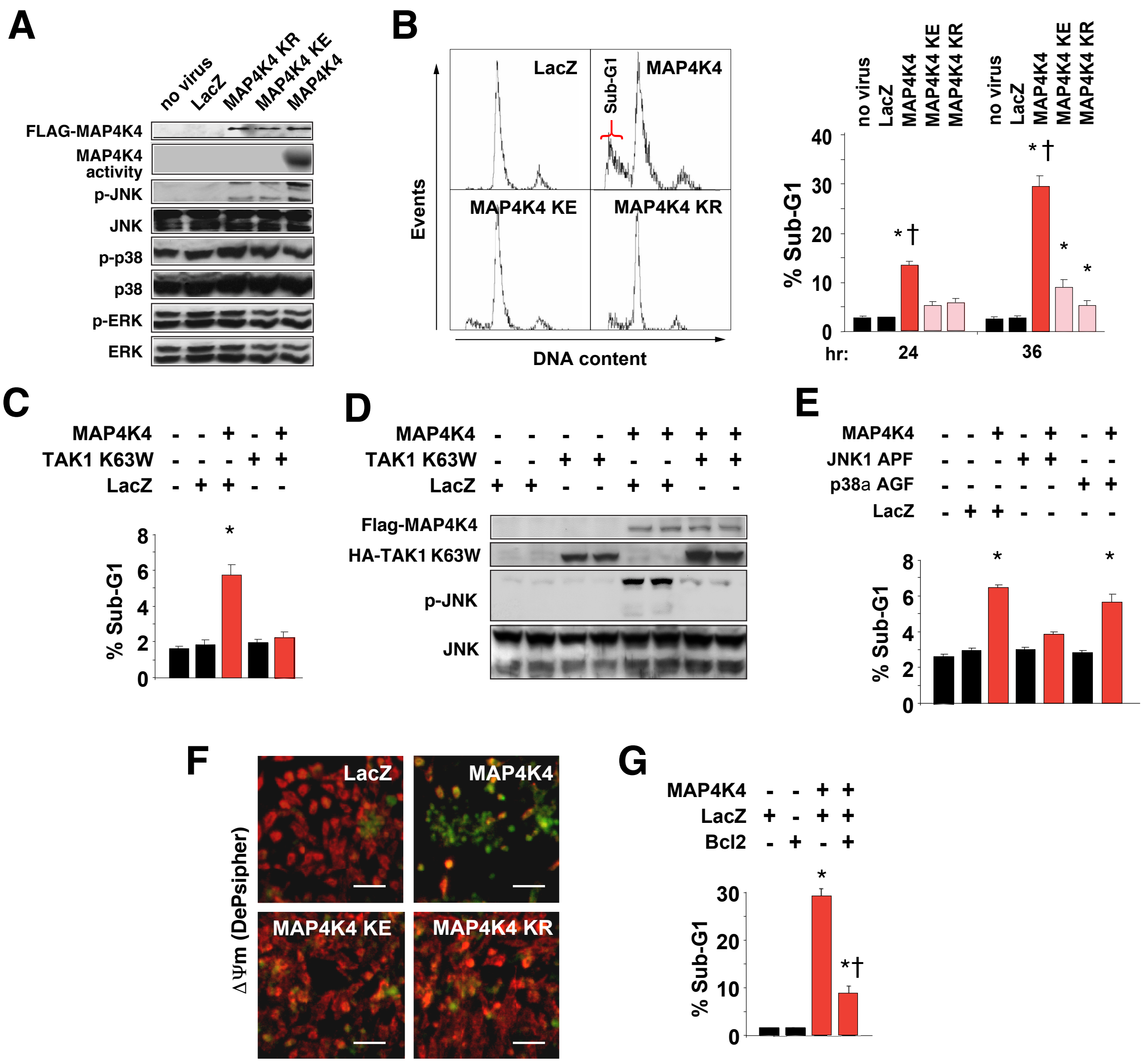

Figure S2, related to Figure 1: Rat cardiomyocyte apoptosis via the MAP4K4-TAK1-JNK mitochondrial death pathway. Rat ventricular myocytes subjected to viral gene transfer were analyzed as shown. (**A**) MAP4K4 activates JNK preferentially. Lysine-substituted mutations of MAP4K4 lack kinase activity yet weakly activate JNK. MAP4K4 activity was measured by the immune complex kinase assay and other end-points by Western blotting. Cells were studied 24 h after infection. (**B**) The lethality of MAP4K4 depends principally on its catalytic activity. Left, Flow cytometry, indicating hypodiploid (sub-G1) DNA. n = 6; *, $P \leq 0.01$ versus LacZ; †, $P \leq 0.01$ for wild-type versus kinase-inactive MAP4K4. Cells were studied 24-36 h after infection. (**C**) Induction of apoptosis by MAP4K4 is blocked by kinase-dead TAK1. n = 6; *, $P \leq 0.01$ versus LacZ. (**D**) Activation of JNK by MAP4K4 is blocked by kinase-dead TAK1. The JNK bands correspond to p54 and p46 splice variants of JNK1/2/3. (**E**) MAP4K4-induced apoptosis is inhibited by kinase-dead JNK1. n = 6; *, $P \leq 0.001$ versus LacZ. (**F**) Dissipation of ΔΨm by MAP4K4, visualized using DePsipher (red → green shift). Bar, 100 μm. (**G**) MAP4K4-induced apoptosis is inhibited by Bcl2. n = 6; *, $P \leq 0.001$ versus LacZ; †, $P \leq 0.03$ versus MAP4K4 alone. In Panels C-G, cells were transduced with blockers for 18 h, then with MAP4K4 for 24 h. All numerical results are shown as the mean ± SE.

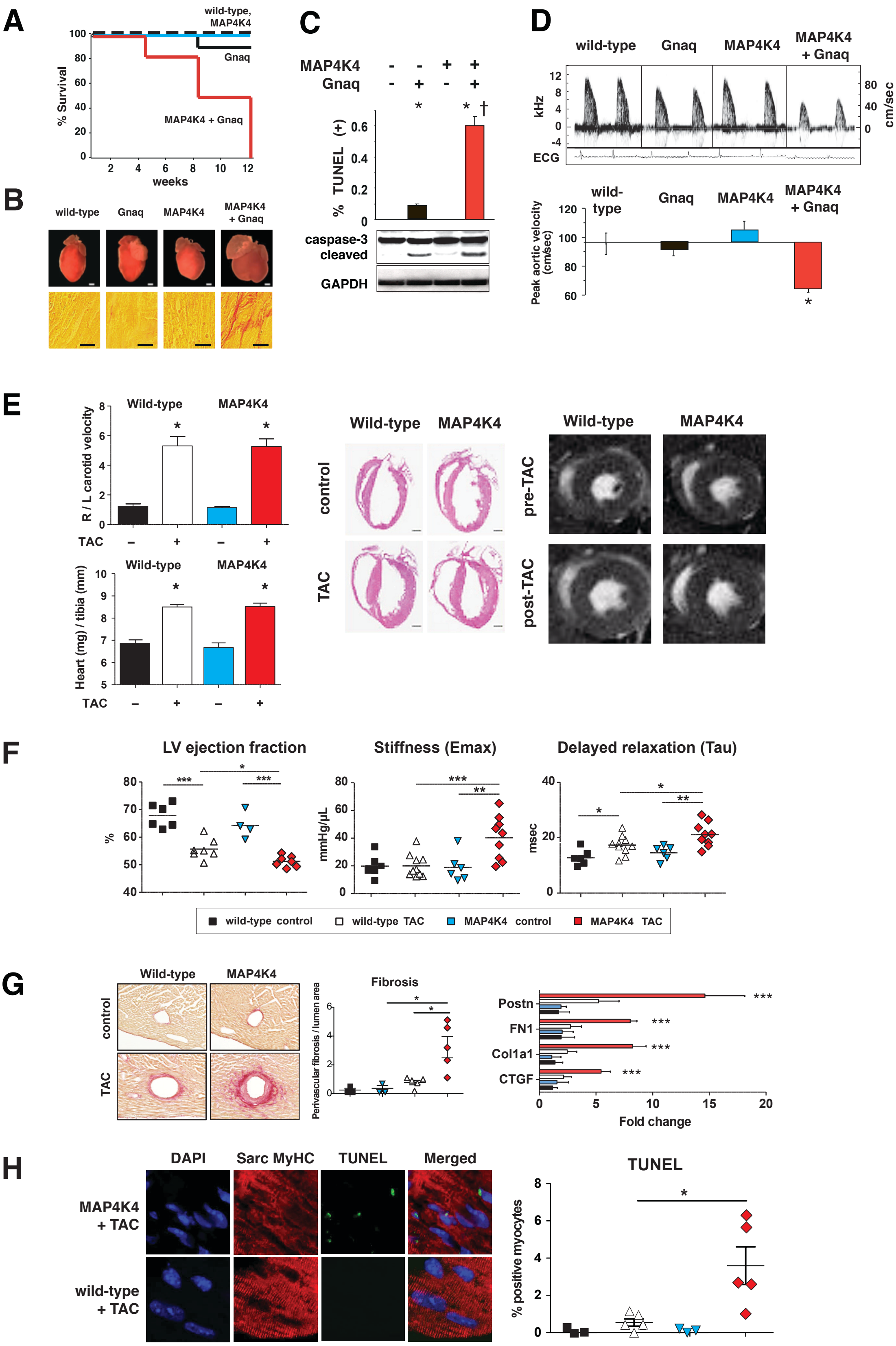
A
wild-type, MAP4K4
Gnaq
MAP4K4 + Gnaq
% Survival
weeks
B
wild-type
Gnaq
MAP4K4
MAP4K4 + Gnaq
C
MAP4K4
Gnaq
% TUNEL (+)
caspase-3 cleaved
GAPDH
D
wild-type
Gnaq
MAP4K4
MAP4K4 + Gnaq
kHz
cm/sec
ECG
Peak aortic velocity (cm/sec)
E
Wild-type
MAP4K4
R / L carotid velocity
TAC
Heart (mg) / tibia (mm)
control
pre-TAC
post-TAC
F
LV ejection fraction
Stiffness (Emax)
Delayed relaxation (Tau)
mmHg/μL
msec
wild-type control
wild-type TAC
MAP4K4 control
MAP4K4 TAC
G
Fibrosis
Perivascular fibrosis / lumen area
Postn
FN1
Col1a1
CTGF
Fold change
H
DAPI
Sarc MyHC
TUNEL
Merged
MAP4K4 + TAC
wild-type + TAC
% positive myocytes

Figure S3, related to Figure 1. MAP4K4 potentiates death signals in mouse myocardium. (**A-D**) Synergy with *Myh6-Gnaq*. (**A**) Survival. Comparable synergy was provoked using each of three independent *Myh6-Map4k4* lines, pooled here. For each genotype, $N \geq 19$; $P < 0.001$ versus single transgenic or non-transgenic littermates**.** (**B)** Top, Marked cardiac enlargement in *Myh6-Map4k4*$^{+/o}$*; Myh6-Gnaq*$^{+/o}$ mice. Bottom, Picrosirius red stain, showing increased LV fibrosis (bottom). Bar, 1 mm (top); 100 μm (bottom). (**C**) Increased TUNEL-positive cardiomyocytes (top) and caspase-3 cleavage (Western blot, bottom). (**D**) Doppler-echocardiography at age 8 wk, showing decreased peak aortic ejection velocity, a measure of ventricular systolic performance, in bi-genic mice. Representative recordings are illustrated at the top. n = 4; *, $P \leq 0.001$. **(E-H)** Synergy with TAC. Mice were 8-10 wk old at the time of aortic banding and were analyzed after 4 wk of increased work load. (**E**) Left to right carotid flow velocity ratio (indicating the degree of constriction) and left ventricular hypertrophy were equal in wild-type and *Myh6-Map4k4* mice. Center, Representative four-chamber hematoxylin and eosin sections. Bar, 2 mm. Right, Representative end-systolic images by 9.4T cine-MRI. (**F**) Cardiac mechanical performance. Left, Depressed left ventricular ejection fraction by cine-MRI. Center, right, Impaired diastolic function, by pressure-volume loop analysis. Results were analyzed by one-way ANOVA and Neuman- Keuls test. (**G**) Increased perivascular fibrosis. Left, Representative images, using picrosirius red. Right, Increased expression of fibrosis-related genes. (**H**) Increased cardiomyocyte apoptosis. Left, Representative TUNEL staining. Cardiomyocytes are visualised using MF20 antibody to sarcomeric MyHC. Colors in panels G, H correspond to the legend in panel F. For MRI, n = 4-7; pressure-volume loops, n = 5-8; histology and qPCR, n = 3-6. *, $P \leq 0.05$; **, $P \leq 0.01$; ***, $P \leq 0.001$. All numerical results are shown as the mean ± SE.

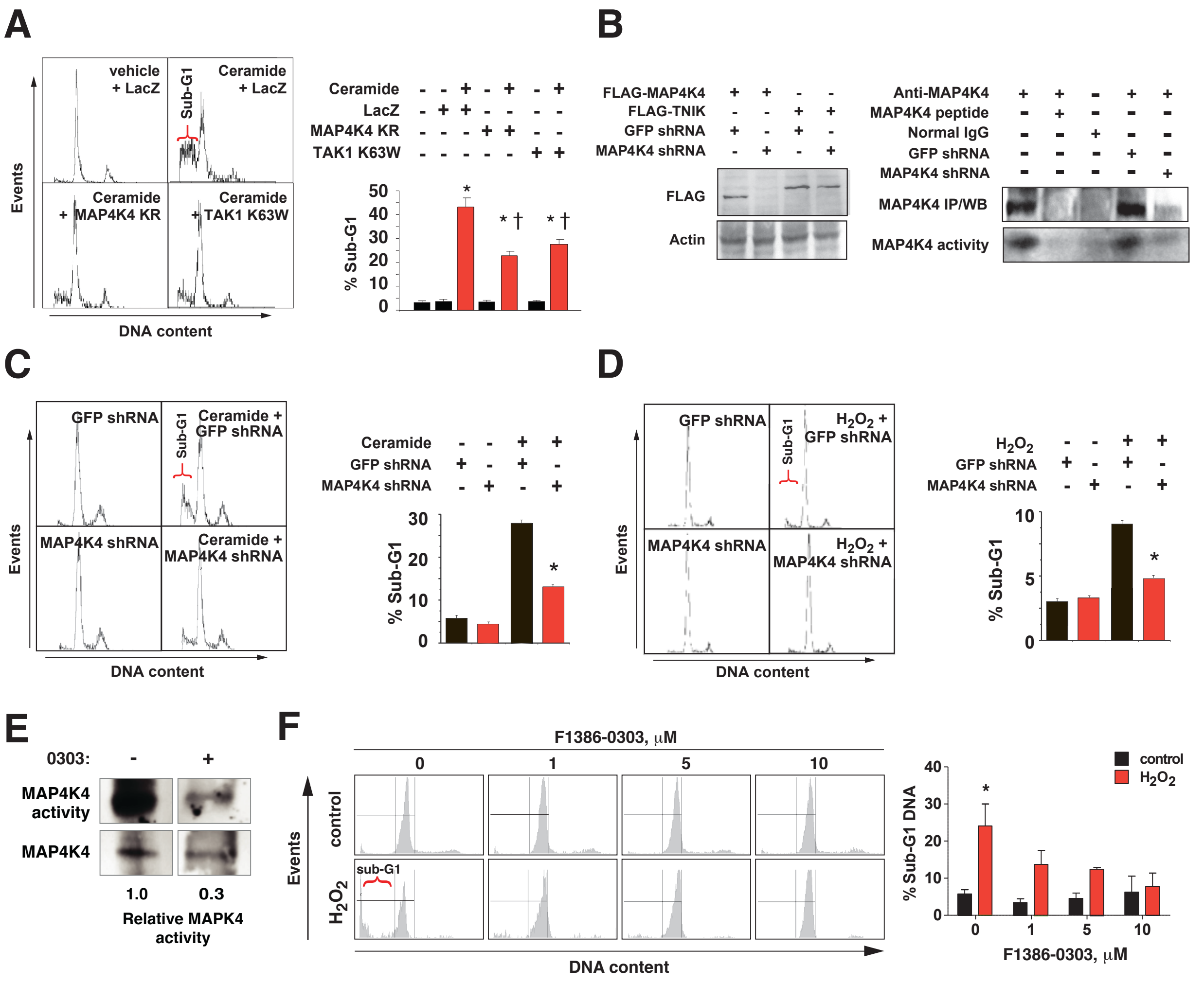

Fig. S4, related to Figure 1. Protection of rat cardiomyocytes by genetic and pharmacological inhibitors of MAP4K4. (**A**) Ceramide-induced apoptosis is inhibited by kinase-inactive mutations of MAP4K4 and TAK1. Left, DNA histograms by flow cytometry. n = 6; *, P ≤ 0.01 versus the LacZ-infected, vehicle-treated negative control; †, P ≤ 0.01 versus the LacZ-infected, ceramide-treated positive control. (**B**) Suppression of MAP4K4 protein and activity by shRNA. Left, Suppression of exogenous MAP4K4 in HEK293T cells by co-transfection of MAP4K4 shRNA. No off-target effects were seen on expression of the MAP4K4 relative TNIK. Right, Suppression of endogenous MAP4K4 expression and activity in rat cardiomyocytes by adenoviral delivery of MAP4K4 shRNA. Upper row, IP/Western blot. Lower row, immune complex kinase activity. (**C**) MAP4K4 shRNA attenuates ceramide-induced apoptosis. Left, DNA histograms by flow cytometry. n = 6; *, P ≤ 0.001. (**D**) MAP4K4 shRNA attenuates H_2O_2-induced apoptosis. Left, DNA histograms by flow cytometry. n = 4; *, P ≤ 0.001. (**E**) In-cell activity of 10 μM F1386-0303 against human MAP4K4 in 293T cells. (**F**) Protection of rat cardiomyocytes by F1386-0303. Left, DNA histograms by flow cytometry. n = 5; *, P ≤ 0.001 versus the vehicle control. All numerical results are shown as the mean ± SE.

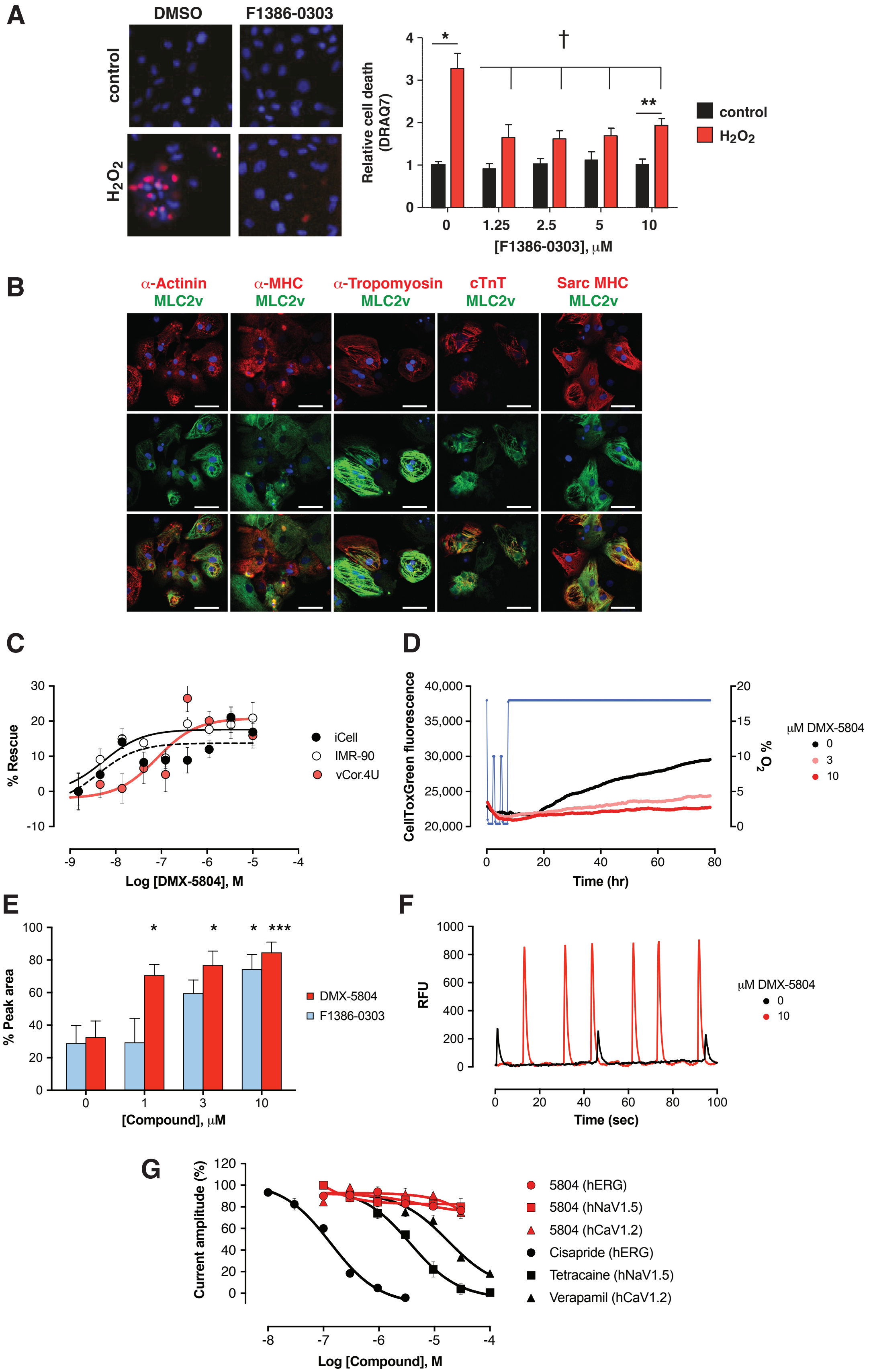
A
DMSO
F1386-0303
control
H_2O_2
Relative cell death (DRAQ7)
*
†
**
0
1.25
2.5
5
10
[F1386-0303], μM
control
H_2O_2
B
α-Actinin MLC2v
α-MHC MLC2v
α-Tropomyosin MLC2v
cTnT MLC2v
Sarc MHC MLC2v
C
% Rescue
Log [DMX-5804], M
iCell
IMR-90
vCor.4U
D
CellToxGreen fluorescence
% O_2
Time (hr)
μM DMX-5804
0
3
10
E
% Peak area
[Compound], μM
DMX-5804
F1386-0303
F
RFU
Time (sec)
μM DMX-5804
0
10
G
Current amplitude (%)
Log [Compound], M
5804 (hERG)
5804 (hNaV1.5)
5804 (hCaV1.2)
Cisapride (hERG)
Tetracaine (hNaV1.5)
Verapamil (hCaV1.2)

Fig. S5, related to Figures 3 and 4. Efficacy and safety of MAP4K4 inhibitors in hiPSC-CMs. (A) Protection of iCell human cardiomyocytes from oxidative stress by F1386-0303, measured as DRAQ7 uptake. Left, Representative microscopy. Red, DRAQ7; blue, Hoechst 33342. n = 3. *, P ≤ 0.05 versus the absence of H_2O_2; **, P ≤ 0.01 versus the absence of H_2O_2; †, P ≤ 0.001 versus H_2O_2 in the absence of F1386-0303. **(B)** Expression of the indicated markers in vCor.4U human ventricular myocytes, by immunostaining. The ventricular chamber-specific protein $MLC2_v$ (green) was expressed in >90% of myocytes identified by the five pan-cardiac markers (red); blue, Hoechst 33342. **C)** Suppression of death from oxidative stress in three independent human cardiomyocyte lines. Cells were assayed in parallel 24 h after treatment with 400 μM H_2O_2 plus DMX-5804 at the indicated concentrations (Cell Titer-Glo viability assay). A representative set of dose-response curves is shown; n = 3. **(D)** Protection of vCor.4U human ventricular myocytes from hypoxia-reoxygenation injury (3 cycles of 0.1% O_2, over a period of 8 h). The extent of cell death induced by this protocol in the absence of compound was 20%. A representative time-course is shown; n = 3. **(E)** Potency of DMX-5804 vs F1386-0303 in preserving spontaneous calcium cycling in vCor.4U human cardiomyocytes, 24 hr after 20 μM menadione; n = 3; *, P ≤ 0.033; ***, P ≤ 0.002. **(F)** Preservation of calcium cycling in vCor.4u cells, 8 hr after hypoxia-reoxygenation injury as in Panel D. Representative tracings are shown; n = 2. **(G)** Ion channel safety screening. Compounds were tested by automated whole-cell patch clamp recording (Patchliner, Nanion Technologies) in stably transfected cells expressing the indicated full length human channels (Chinese hamster ovary: hERG, hNaV1.5; human embryonic kidney: hCaV1.2). IC_{50} values (μM) were obtained from a 4-parameter logistic fit of the concentration-response data. hERG: Cisapride, 0.12; 5804, > 30. hNav1.5: Tetracaine, 3.2; 5804: > 30. hCaV1.2: Verapamil 16.7; 5804, > 30; n ≥ 16. All numerical results are shown as the mean ± SE.

Table S1, related to Figure 1. Serial non-invasive imaging demonstrates a synergistic effect of MAP4K4 and biomechanical stress on LV ejection fraction.

Genotype	Time	Wild-type	Wild-type	*Myh6-Map4k4*	*Myh6-Map4k4*
**TAC**		**-**	**+**	**-**	**+**
Heart rate (bpm)	pre	486 ± 12	523 ± 18	528 ± 13	519 ± 16
	post	522 ±11	513 ± 19	491 ± 10	503 ± 18
End systolic volume (μl)	pre	19 ± 1	20 ± 2	22 ± 0	19 ± 2
	post	20 ± 1	31 ± 1***	24 ± 1	34 ± 1***
Stroke volume (μl)	pre	39 ± 2	42 ± 1	43 ± 2	40 ± 2
	post	42 ± 1	39 ± 1	44 ± 2	36 ± 1**
Ejection fraction (%)	pre	67 ± 1	67 ± 2	66 ± 2	68 ± 2
	post	68 ± 2	56 ± 2***	64 ± 2	51 ± 2***†
Cardiac output (ml/min)	pre	18 ± 1	22 ± 1	22 ± 1	21 ± 2
	post	22 ± 1	19 ± 1**	22 ± 1	18 ± 1**
Cardiac index (ml/min/mg)	pre	1.50 ± 0.09	1.28 ± 0.07	1.22 ± 0.05	1.32 ± 0.09
	post	1.50 ± 0.08	1.53 ± 0.04*	1.28 ± 0.06	1.54 ± 0.57*
Left ventricular mass (mg)	pre	99 ± 4	109 ± 3	106 ± 4	107 ± 4
	post	113 ± 3	156 ± 5***	114 ± 4	164 ± 4***
Body weight (g)	pre	26 ± 0	26 ± 0	25 ± 0	25 ± 0
	post	27 ± 1	26 ± 1	27 ± 1	25 ± 1
LV mass / body weight	pre	3.87 ± 20	4.27 ± 16	2.48 ± 17	4.28 ± 17
	post	4.28 ± 21	6.04 ± 31***	4.20 ± 17	6.47 ± 20***

Results of cine-MRI are shown as the mean ± SEM for *Myh6-Map4k4* mice and littermate controls, before and 4 wk after TAC. Asterisks, TAC versus sham-operated mice: * $p < 0.05$, ** $p < 0.01$, *** $p < 0.001$. †, *Myh6-Map4k4* TAC versus wild-type TAC, $p < 0.05$.
